# Enhanced quantitation of pathological α-synuclein in patient biospecimens by RT-QuIC seed amplification assays

**DOI:** 10.1371/journal.ppat.1012554

**Published:** 2024-09-20

**Authors:** Ankit Srivastava, Qinlu Wang, Christina D. Orrù, Manel Fernandez, Yaroslau Compta, Bernardino Ghetti, Gianluigi Zanusso, Wen-Quan Zou, Byron Caughey, Catherine A. A. Beauchemin

**Affiliations:** 1 Laboratory of Neurological Infections and Immunity, Rocky Mountain Laboratories, Division of Intramural Research, National Institute of Allergy and Infectious Diseases (NIAID), National Institutes of Health (NIH), Hamilton, Montana, United States of America; 2 Bioinformatics and Computational Biosciences Branch, National Institute of Allergy, and Infectious Diseases (NIAID), National Institutes of Health (NIH), Bethesda, Maryland, United States of America; 3 Parkinson’s Disease & Movement Disorders Unit, Neurology Service, Hospital Clínic I Universitari de Barcelona; IDIBAPS, CIBERNED (CB06/05/0018-ISCIII), ERN- RND, Institut Clínic de Neurociències (Maria de Maeztu Excellence Centre), Universitat de Barcelona. Barcelona, Catalonia, Spain; 4 Indiana University School of Medicine, Indianapolis, Indiana, United States of America; 5 Department of Neurosciences, Biomedicine and Movement Sciences, University of Verona, Verona, Italy; 6 Departments of Pathology and Neurology, Case Western Reserve University School of Medicine, Cleveland, Ohio, United States of America; 7 Institute of Neurology, Jiangxi Academy of Clinical Medical Sciences, The First Affiliated Hospital, Jiangxi Medical College, Nanchang University, Nanchang, Jiangxi Province, China; 8 Department of Physics, Toronto Metropolitan University, Toronto, Canada; 9 Interdisciplinary Theoretical and Mathematical Sciences (iTHEMS) at RIKEN, Wako, Japan; Colorado State University College of Veterinary Medicine and Biomedical Sciences, UNITED STATES OF AMERICA

## Abstract

Disease associated pathological aggregates of alpha-synuclein (αSyn^D^) exhibit prion-like spreading in synucleinopathies such as Parkinson’s disease (PD) and dementia with Lewy bodies (DLB). Seed amplification assays (SAAs) such as real-time quaking-induced conversion (RT-QuIC) have shown high diagnostic sensitivity and specificity for detecting proteopathic αSyn^D^ seeds in a variety of biospecimens from PD and DLB patients. However, the extent to which relative proteopathic seed concentrations are useful as indices of a patient’s disease stage or prognosis remains unresolved. One feature of current SAAs that complicates attempts to correlate SAA results with patients’ clinical and other laboratory findings is their quantitative imprecision, which has typically been limited to discriminating large differences (e.g. 5–10 fold) in seed concentration. We used end-point dilution (ED) RT-QuIC assays to determine αSyn^D^ seed concentrations in patient biospecimens and tested the influence of various assay variables such as serial dilution factor, replicate number and data processing methods. The use of 2-fold versus 10-fold dilution factors and 12 versus 4 replicate reactions per dilution reduced ED-RT-QuIC assay error by as much as 70%. This enhanced assay format discriminated as little as 2-fold differences in αSyn^D^ seed concentration besides detecting ~2-16-fold seed reductions caused by inactivation treatments. In some scenarios, analysis of the data using Poisson and midSIN algorithms provided more consistent and statistically significant discrimination of different seed concentrations. We applied our improved assay strategies to multiple diagnostically relevant PD and DLB antemortem patient biospecimens, including cerebrospinal fluid, skin, and brushings of the olfactory mucosa. Using ED αSyn RT-QuIC as a model SAA, we show how to markedly improve the inter-assay reproducibility and quantitative accuracy. Enhanced quantitative SAA accuracy should facilitate assessments of pathological seeding activities as biomarkers in proteinopathy diagnostics and prognostics, as well as in patient cohort selection and assessments of pharmacodynamics and target engagement in drug trials.

## Author summary

Seed amplification assays (SAAs) such as RT-QuIC detect pathological α-Syn aggregates with prion-like self-propagating (seeding) activity from various tissue biospecimens of synucleinopathy patients. However, clinical applications of current SAAs to neurodegenerative diseases can be hampered by their quantitative imprecision in stratifying levels of pathological seeds as biomarkers. In this study, we tested the influence of various assay variables including dilution factor, number of replicates, and quantitation methods in end-point dilution RT-QuIC (ED RT-QuIC) using PD and DLB patient samples, specifically brain tissue, CSF, skin, and nasal brushings. Our study highlights how assay design can markedly improve seed quantification in clinical samples. Better proteopathic seed quantification should enable more precise evaluation of pathological seeding activity to support important clinical and research applications.

## Introduction

Synucleinopathies such as Parkinson’s disease (PD) and dementia with Lewy bodies (DLB) and multiple system atrophy display varied clinical phenotypes and their diagnosis relies heavily on the onset of distinct clinical symptoms and brain imaging profiles [1,2]. Frequent misdiagnosis or delayed diagnosis is often reported owing to phenotypic overlaps with other neurodegenerative disorders and a paucity of definitive biomarkers [3–7].

However, one promising, and presumably etiological, biomarker is disease-associated α-Synuclein aggregates (αSyn^D^) in the central nervous system, bodily fluids (e.g., CSF) and certain peripheral tissues (e.g. skin, OM, submandibular glands, and saliva) as detected by seed amplification assays (SAAs) [8–15]. SAAs such as RT-QuIC exploit the ability of αSyn^D^ to induce the conversion of a vast stoichiometric excess of recombinant αSyn monomers (i.e., the assay substrate) into amyloid fibrils that become readily detectable using an amyloid-sensitive fluorophore, Thioflavin T (ThT). These assays are performed in multi-well plates that are shaken and continuously monitored for ThT fluorescence in fluorescence plate readers as reactions progress [16,17].

Accordingly, SAAs are emerging as useful diagnostic and prognostic tools. Indeed, multiple recent studies have detected αSyn^D^ even in early prodromal stages of synucleinopathy such as in idiopathic REM sleep behavioural disorder (iRBD) or pure autonomic failure [18,19]. Although moderate correlations of SAA-derived quantitation of proteopathic seeding with patient associated clinical scores have been reported, it is still unclear to what degree relative levels of αSyn^D^ seeding activity in a given biospecimen could serve as an index of disease severity and progression (e.g., [8,20,21]). If a robust link can be established between αSyn^D^ level and disease severity, then quantification of αSyn^D^ levels as a pharmacodynamic biomarker could subsequently serve as surrogate markers of response (or lack of thereof) to experimental drugs in clinical trials or eventually even in clinical practice.

Previous reports have demonstrated two major approaches for quantifying proteopathic seed load (e.g. PrP^Sc^, αSyn^D^) in biospecimens using RT-QuIC assays: endpoint dilution (ED) and lag-time based approaches. In the latter, seed concentration is deduced from a calibration curve created by the measurement of lag times, i.e., reaction times required to reach a selected fluorescence threshold, generated by known relative quantities of proteopathic seeds [22,23]. For example, this approach has been used to show a log-linear relationship between seed concentration and αSyn^D^ seeding activity in *postmortem* ventricular CSF which in turn correlated with reduced survival in DLB patients [24]. However, much lower seeding activities have been reported for CSF collected from living patients by lumbar puncture (e.g. [9]), making lag phases more erratic and quantitation more challenging. Lag times can also be influenced by differences in sample manipulation, matrix composition, or other factors in addition to the initial seed concentration [25], and such factors may vary markedly between individual patients’ biospecimens, and the matrix used for the calibration curve.

**Fig 1:**
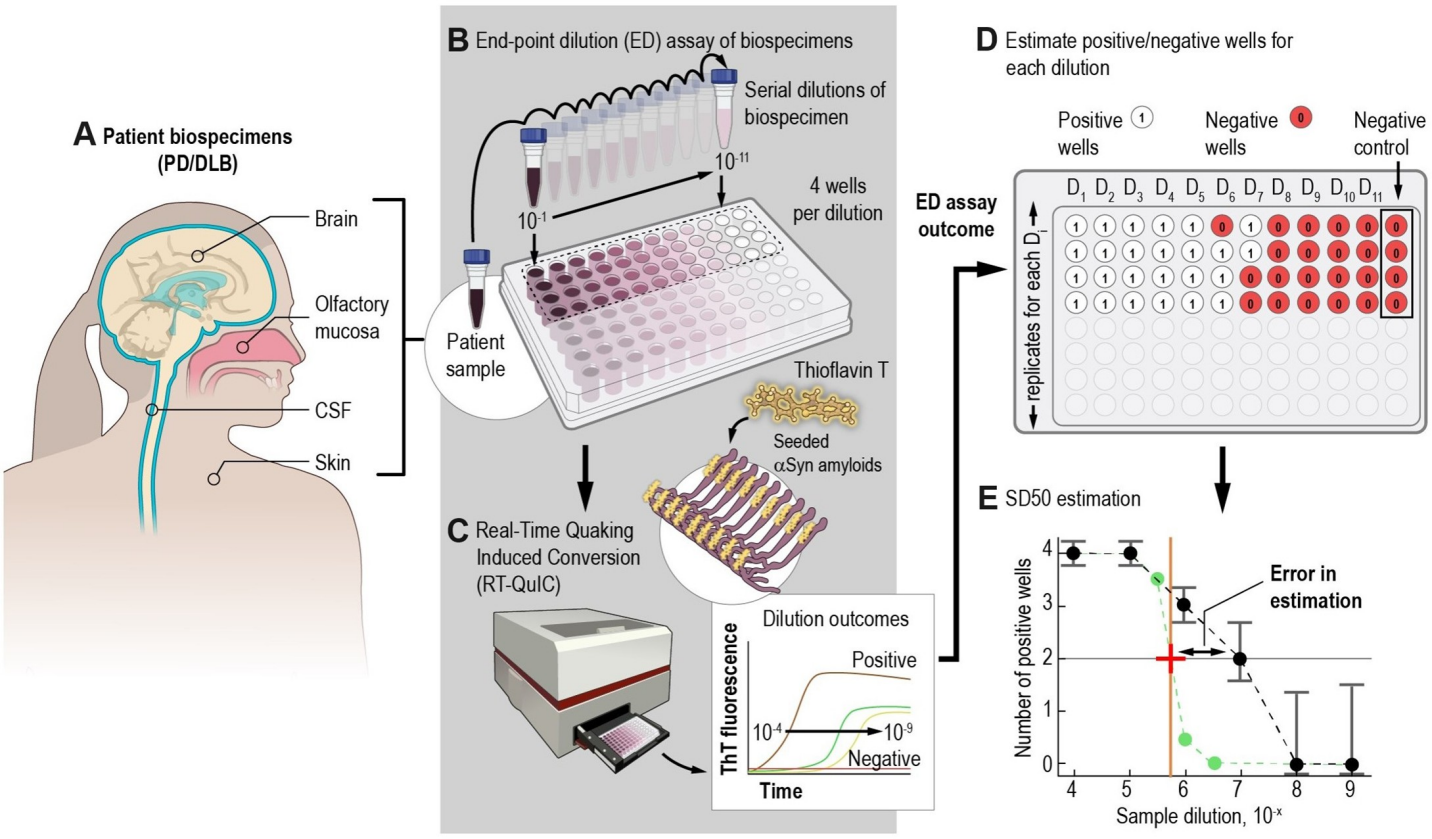
Schematic diagram of end-point dilution (ED) RT-QuIC assay for quantifying pathological αSyn^D^ seeds. (A) Various type of PD/DLB patient samples for quantitating αSyn^D^ seeds. (B) 10-fold serial dilutions of tissue/biospecimens were prepared and seeded (4-replicates/dilution) in a 96-well plate containing reaction mix comprising buffer components, recombinant αSyn substrate and detection dye Thioflavin-T (ThT). (C) The plates were incubated under standard αSyn RT-QuIC conditions and resulting outcomes plotted as ThT fluorescence versus time. (D) Positive replicate wells per dilution were enumerated based on whether the ThT fluorescence threshold was reached by a selected cut-off time. The concentration of pathological αSyn^D^ seeds from patient biospecimen is expressed as SD50 (50% seeding dose) per volume or mass of sample, based on the dilution at which 50% of wells were ThT-positive, as estimated using the Spearman-Karber or other analytical methods. (E) Individual biospecimens tested in independent ED assays have often displayed variability in ThT-positivity, resulting in deviations of the SD50 estimates from the true value (red plus).

ED RT-QuIC provides a complementary, or alternative, approach to quantitation that depends primarily on how seeds dilute out and is less exclusively dependent on the relative kinetics with which those seeds induce fibrilization of the substrate. In ED RT-QuIC, the seeding “dose” or amount of sample that gives 50% ThT positive replicate wells (SD50) is estimated. SD50 is analogous to the 50% lethal dose (LD50) or 50% tissue culture infectious dose (TCID50) estimation that is widely employed in cell culture and animal bioassays [26]. Thus far, ED RT-QuIC assays have typically relied on the Spearman-Kärber (SK) method to estimate the SD50 by results of replicate reactions seeded with serial dilutions of biospecimens (Fig 1) [17,27–30]. ED-based quantitation provides a potential means of testing large patient cohorts and multi-center studies. Of particular interest, two past studies utilizing CSF and skin samples from independent PD patient cohorts have shown moderate correlations between the SD50 concentrations and patients’ age and disease duration [11,20], but other studies have failed to show such correlations [8,31]. ED-based seed quantification also has been successfully applied in a microfluidic system [32].

Nonetheless, ED-based SAA quantification can be negatively influenced by factors such as inefficient or non-homogeneous seed dilution (e.g., due to aggregation and/or losses to tubes and pipette tips), variations in substrate quality, buffer composition, and sample components [33]. Besides, the commonly used SK-based estimation method does not provide the most accurate estimation of the SD50 from sample biospecimens [34–38]. The present study examines effects of assay design choices, notably dilution factor, replicate number, and data analysis methods, on the reproducibility and statistical robustness of ED SAA-based estimations of seed concentration in various biospecimens, specifically brain tissue, CSF, skin, and OM, in the context of αSyn RT-QuIC.

## Results

### Influence of assay design variables on seed quantitation in ED RT-QuIC assays

**Fig 2:**
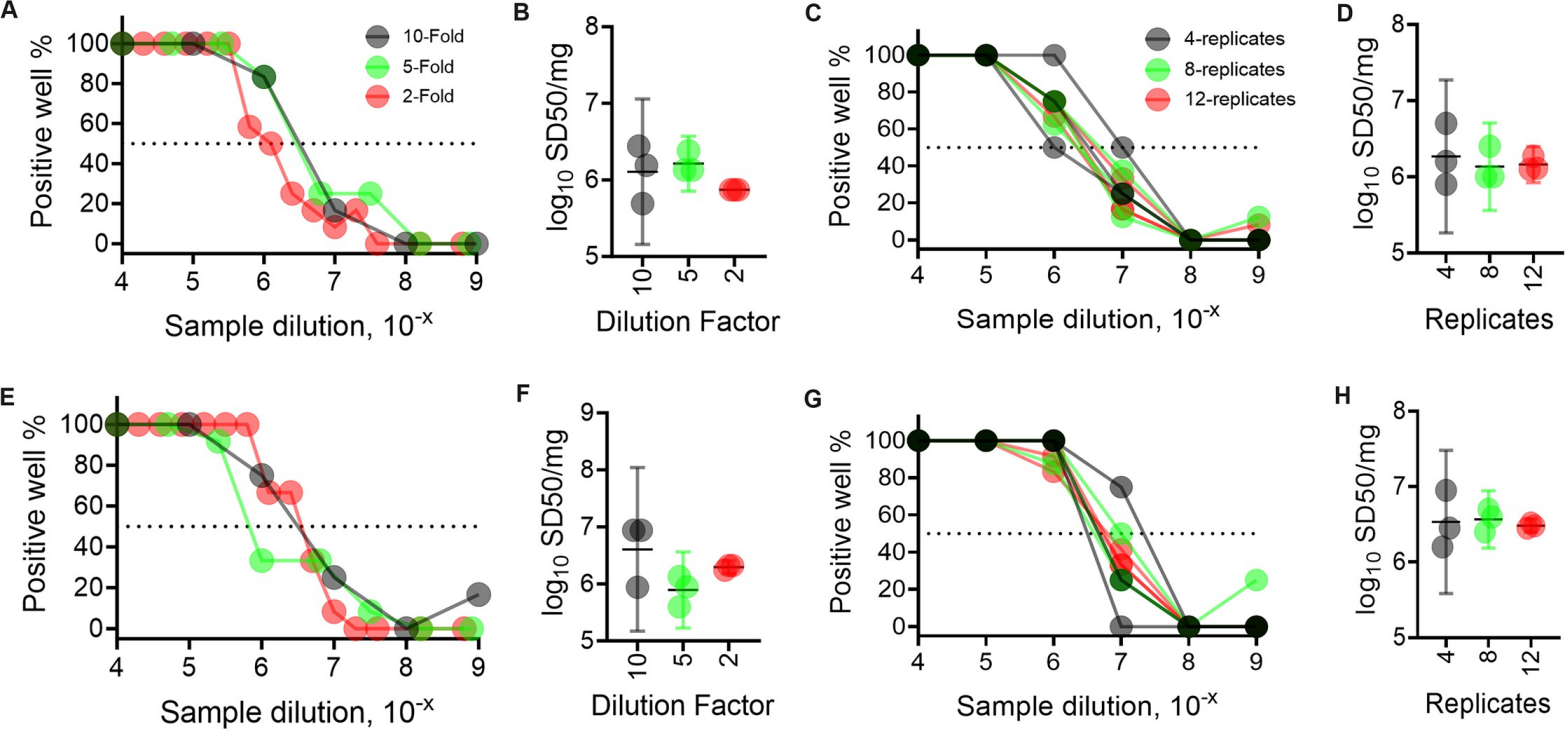
Effect of dilution factor and replicate number on the reproducibility of αSyn^D^ seed concentration estimates in PD (A-D) and DLB (E-H) brain homogenates by ED RT-QuIC assays. (A) & (E) Percent positive wells for triplicate assays versus sample dilution with 10-, 5-, and 2-fold dilution factors (as indicated in color legend). (B) & (F) Effect of decreasing dilution factor on consistency of log_10_ SD50/mg estimates from 3 independent ED assays. (C) & (G) Percent positive wells from triplicate ED assays versus sample dilution with 4-, 8-, and 12-replicates per dilution (as indicated in color legend). (D) & (H) Effect of increasing replicate numbers on consistency of log_10_SD50/mg estimates from triplicate assays. Data points in A, E correspond to arithmetic averages while C, G represent individual replicates of the total % positive wells from the 3 separate assays, each with quadruplicate wells at each dilution. Horizontal dotted lines in A, C, E, G denote 50% positive wells. Data points in B, F, D, H indicate log_10_ SD50/mg brain tissue, with horizontal and vertical bars indicating the mean and 95% CI respectively.

As a model SAA, we used ED αSyn RT-QuIC to quantify pathological αSyn^D^ seed concentration. We started with a conventional analysis of brain homogenates (BHs) from neuropathologically confirmed PD and DLB cases using 10-fold serial dilutions, 4 replicate wells per dilution, and SK analysis of the proportion of ThT-positive wells at each dilution to estimate the concentration of αSyn^D^ SD50 seeding units in the original brain tissue (Fig 1) [9,29]. We then compared the results to those obtained using decreased dilution factors and increased replicate wells at each dilution, each of which has been shown to reduce variability in analogous viral load quantitation [39]. A much higher number of outcomes (colored circles) per ED assay were obtained with narrower dilution factors (Figs 2A, 2E and S1). This increased the likelihood that 50% of the positive wells in an ED assay would be more accurately identified (horizontal broken line, Fig 2A and 2E) thereby reducing variability in SD50 estimates (lower 95% CI, Fig 2B and 2F). Indeed, smaller sample dilution intervals, i.e., 5- and 2-fold as opposed to 10-fold reduced the standard error (SE, see Methods) of log_10_ SD50/mg estimates from 3 separate assays up to ~20% and ~60%, respectively (S2A and S2C Fig).

Similarly, increasing the number of replicate wells per dilution from 4 to 8 or 12 in 10-fold dilution series helped in obtaining more converged data points (due to better averaging of outcomes per dilution) that further improved the consistency of correctly identifying 50% positive wells (colored circles, Fig 2C and 2G). This also helped in reducing the variability in log_10_ SD50 /mg estimates from triplicate ED assays (lower 95% CI, Fig 2D and 2H). Besides, the decreases in SE of log_10_ SD50 /mg estimates by increasing replicate numbers were comparable to the effect of decreasing dilution factors (S2B and S2D Fig).

We next investigated the combined impact of increasing replicate numbers in an ED assay using a 2-fold dilution series. We observed further improvement in data point convergence and reduction in variability for log_10_ SD50 /mg estimates with higher replicates per dilution (S3 Fig). Interestingly, SE of 2-fold dilutions with 4 replicates was comparable to the conventional 10-fold dilutions with 12 replicate wells per dilution (S2B and S2D Fig). Further increases in replicate number per dilution from 8 to 12 for the PD or DLB samples yielded >70% reduction in SE of the mean log_10_ SD50/mg estimates.

### Assessment of other seed concentration estimation methods with ED RT-QuIC data

The SK method has been reported to yield systematic 23% overestimations (10^+0.09^ SD50/mg) of the median tissue culture infective dose in virus titrations [35]. Furthermore, the approximations used in the SK method rely on theoretical expectations that the number of positive wells should decrease monotonically with increasing dilutions. As assay data deviate from this theory, SK-based approximations become increasingly inaccurate, resulting in larger deviations from true values [39]. Thus, we next evaluated the performance of three alternative ED analysis methods: Reed-Muench (RM); a least-square fit hereafter referred to as the Poisson model (Poisson); and midSIN (**m**easure of **i**nfectious **d**ose in **S**pecific **In**fections) (see Methods for details).

**Fig 3:**
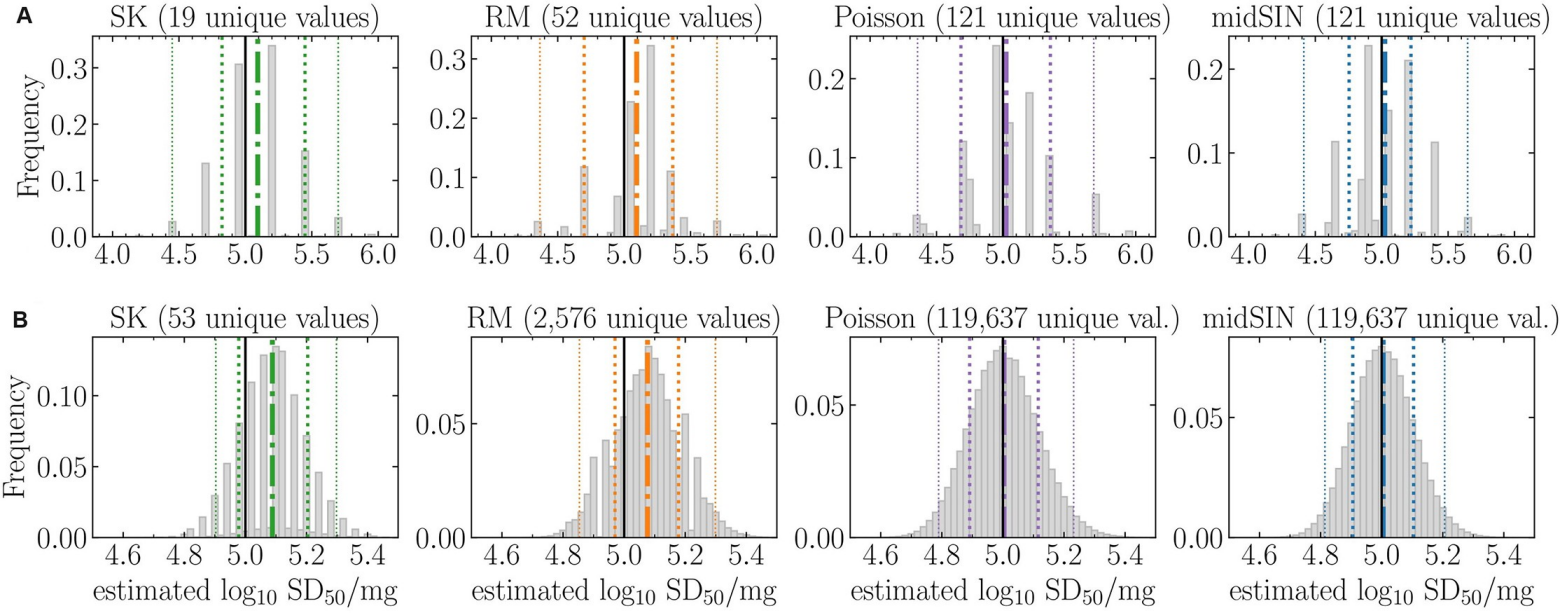
Theoretical comparison of various SD50 estimation methods in 10F4R and 2F8R assays. For a hypothetical sample with a seed concentration of 10^5^ SD50/mg, one million random plates were generated, with each plate having a certain number of positive wells (ThT signals above threshold) in each dilution. For each outcome, the SK, RM, Poison or midSIN, methods were used to estimate the log_10_ SD50/mg αSyn^D^ seed concentration for either (A) the 10F4R assay with an initial dilution of 10^−4^, 5 dilutions separated by 10-fold, and 4 replicates/dilution, resulting in 121 unique readout outcomes; or (B) the 2F8R assay with an initial dilution of 10^−4^, 12 dilutions separated by 2-fold, and 8 replicates/dilution, resulting in 119,637 unique readout outcomes. Depending on the estimation method, different readout outcomes can result in the same estimated log_10_ SD50/mg. The number of unique concentration estimates obtained by each method is indicated above each graph. The true sample concentration (10^5^ SD50/mg) (vertical solid black line), along with the mean (dash-dot), 68% CI (thick dotted), and 95% CI (thin dotted) of the estimated concentrations are indicated.

We simulated one million random ED assay readout outcomes (number of positive replicate wells at each dilution, see Methods) for a hypothetical sample with a known αSyn^D^ seed concentration (10^5^ SD50/mg) to compare the performance of the 4 estimation algorithms in a conventional 10F4R [10-fold (10F) dilution, 4 replicates (4R) at each of 5 dilutions using a total of 20 wells] and an alternative 2F8R [2-fold (2F) dilution, 8 replicates (8R) at each of 12 dilutions using 96 wells] ED assay format (Fig 3).

The alternative 2F8R format makes use of 4.8 times more wells and given this increased amount of information was expected to capture smaller differences in sample seed concentrations. For example, at a seed concentration of 10^5^ SD50/mg, the 4 most likely ED assay outcomes in the 2F8R format, namely [8, 8, 8, 7, 5, 3, 1, 0, 0, 0, 0, 0], [8, 8, 8, 7, 5, 2, 1, 0, 0, 0, 0, 0], [8, 8, 8, 7, 5, 3, 1, 1, 0, 0, 0, 0] and [8, 8, 8, 7, 4, 3, 1, 0, 0, 0, 0, 0] positive wells out of 8 replicates at each dilution, would all be represented as [4, 3, 0, 0, 0] positive wells out of 4 replicates in the 10F4R format. This is because the reduced number of replicates and more widely spaced dilutions in the 10F4R format reduce the diversity of outcomes that can be expressed by the assay. In fact, the diversity of the randomly generated assay outcomes increased from 121 in 10F4R to 119,637 with 2F8R, i.e. the 4.8-fold increase in the number of wells used increased assay outcome diversity by over 900-fold.

But some of the methods used to estimate the SD50 seed concentration from an ED assay outcome can effectively reduce this diversity, and hence the accuracy of the SD50 estimate. The 121 different assay outcomes obtained with the conventional 10F4R ED format (Fig 3A) were reduced to only 19 different SK-estimated SD50 concentrations, i.e. several differences in the number of positive wells at each dilution (e.g., [4, 3, 0, 0, 0] and [4, 2, 1, 0, 0]) resulted in the same SK-estimated seed concentration (e.g., 4.949 log_10_ SD50/mg), visible as large gaps between histogram bars (Fig 3A). Overall, SK estimated ~3 times fewer unique SD50 values compared to the RM estimation, and ~6 times fewer than the Poisson and midSIN estimations (Fig 3A). The effect was more pronounced in the 2F8R format, wherein SK estimated >2,000 times fewer unique SD50 values (53 compared to 119,637) than the Poisson and midSIN methods, thus reducing the accuracy of the SD50 estimates (Fig 3B). This means that the SK method is less sensitive to small changes in seed concentration as it fails to capture assay outcome differences that other methods can distinguish. The SK method had a degeneracy (range of concentrations that map to the same estimated concentration) on the order of ±0.1 log_10_ SD50/mg and ±0.01 log_10_ SD50/mg in the 10F4R and 2F8R formats, respectively, compared to ±0.001 log_10_ SD50/mg and ±10^−6^ log_10_ SD50/mg for estimation by Poisson and midSIN. Additionally, as expected, both the SK and RM methods overestimated the SD50 by 23% (+0.09 log_10_ SD50/mg), irrespective of the assay format.

In summary, the alternative 2F8R format increased the accuracy of the estimated SD50 (95% CI reduced from ±1.0 log_10_ SD50/mg to ±0.3 log_10_ SD50/mg), irrespective of the estimation method used, due to both smaller fold dilution (2-fold versus 10-fold) and higher number of replicate wells per dilution (8 rather than 4). While the SK method is less sensitive (resolution accuracy of ±0.1 log_10_ SD50/mg in 10F4R and ±0.01 log_10_ SD50/mg in 2F8R) than the RM, which itself is less sensitive than Poisson and midSIN methods, the SK method’s lack of resolution accuracy is less than, and therefore insignificant compared to, the physical accuracy of the assays themselves (±1.0 to ±0.3 log_10_ SD50/mg, respectively), with the current 10F4R and 2F8R assay formats. Nonetheless, the overestimation of the SD50 by the SK and RM methods, and the increased robustness of the midSIN method [39] make midSIN a preferable choice, especially in scenarios in which still larger numbers of replicate reactions per sample dilution become feasible e.g.: microfluidic set ups such as one described recently [32].

### Development and reproducibility assessment of an alternative quantitation framework

Based on the observations reported above, we devised an alternative ED assay framework to improve seed distribution, the robustness of assay outcomes, and the SD50 estimation. This alternative ED assay for BH samples differed from our conventional ED assay (10-fold dilutions and 4 replicates, hereafter “10F4R”) in the following ways: First, a pre-sonication step was implemented; second, samples were diluted in PBS/N2 with 0.0001% non-synucleinopathy BH to reduce seed aggregation and losses to surfaces (see Methods); third, 2-fold serial dilutions, with 8 replicates per dilutions (hereafter “2F8R”) was used (here, 8 replicates, rather than the more optimal 12, was chosen for practical reasons, e.g., availability of biospecimens, substrate and plate readers); and fourth, an alternative (though mathematically equivalent) method was used to determine well positivity based on ThT fluorescence (see Methods for details). We then compared the estimated SD50s for various BH samples using the 10F4R and 2F8R ED assays.

**Fig 4:**
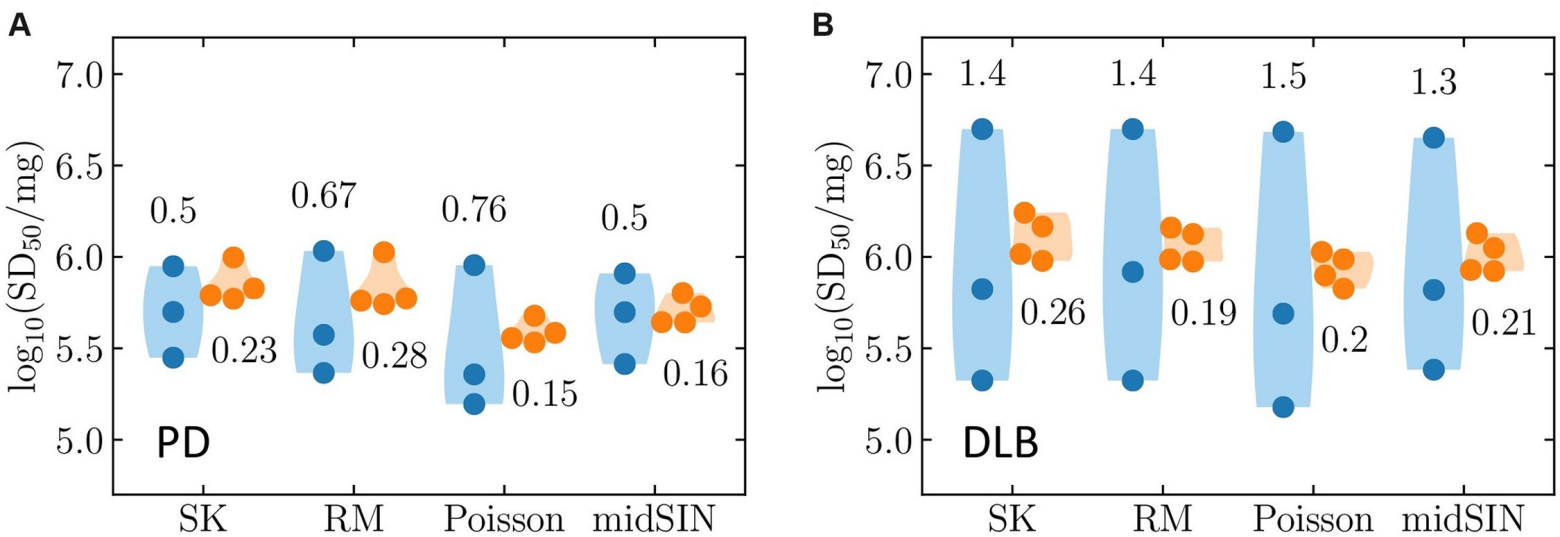
Reproducibility assessment of the 10F4R and 2F8R assay in estimating αSyn^D^ seed concentrations. (A) & (B) Replicate heterogeneity in log_10_ SD50/mg values obtained from ED assays performed in triplicate on PD and DLB BHs, respectively. The 2F8R assay (orange) reduces variability in SD50 estimates compared to the 10F4R assay (blue). Comparison of 4 quantification algorithms displays enhanced quantification accuracy of Poisson and midSIN (low CIs) in the 2F8R assay. Each colored circle represents one of the 3 or 4 replicate measurements, the numbers correspond to the difference between the minimum and maximum log_10_ SD50/mg values obtained, and the shading indicates this [min., max.] range.

As expected, we observed greater variability in SD50 concentration estimates from 3–4 independent assays for both PD and DLB BHs analyzed in the 10F4R format compared to those obtained with the 2F8R format (Fig 4A and 4B respectively). One issue seen in both assays was occasional, most likely false-positive, wells at the largest dilutions (S4A and S4C Fig).

While this issue affected both the 10F4R and 2F8R assays, the larger number of measurements in the latter helped average over that apparent noise and thus provided more robust averaged dilution profiles. While SD50s estimated by the RM and SK methods tended to be higher than estimates by the Poisson and midSIN methods (S4B and S4D), all four methods performed comparably in this pair of samples across multiple independent assays of our PD and DLB brain tissue samples (Fig 4). Since the RM and SK methods tend to overestimate the SD50, the latter two methods should be preferred. Generally, midSIN might provide a slight advantage over the Poisson method because the latter relies on performing a fit to the data, which can occasionally fail to identify the best-fit, whereas the former is merely the result of performing a computation (see Methods for details). In general, the 2F8R assay format helped in reducing the variability and obtaining reproducible SD50 estimates from both PD and DLB samples.

### Improved stratification of samples differing in αSyn seed concentrations

Conventional SD50 estimates using patients’ CSF specimens have shown only moderate, if any, correlations with age and disease duration in PD patients at advanced disease stages [11,20]. Perhaps, an ability to discriminate smaller differences in αSyn^D^ seeding could enhance the utility of SAAs in stratifying various clinical stages of synucleinopathies. With this possibility in mind, two approaches were used to compare the performance of the 10F4R and 2F8R assays in distinguishing smaller increments in seeding activity.

**Fig 5:**
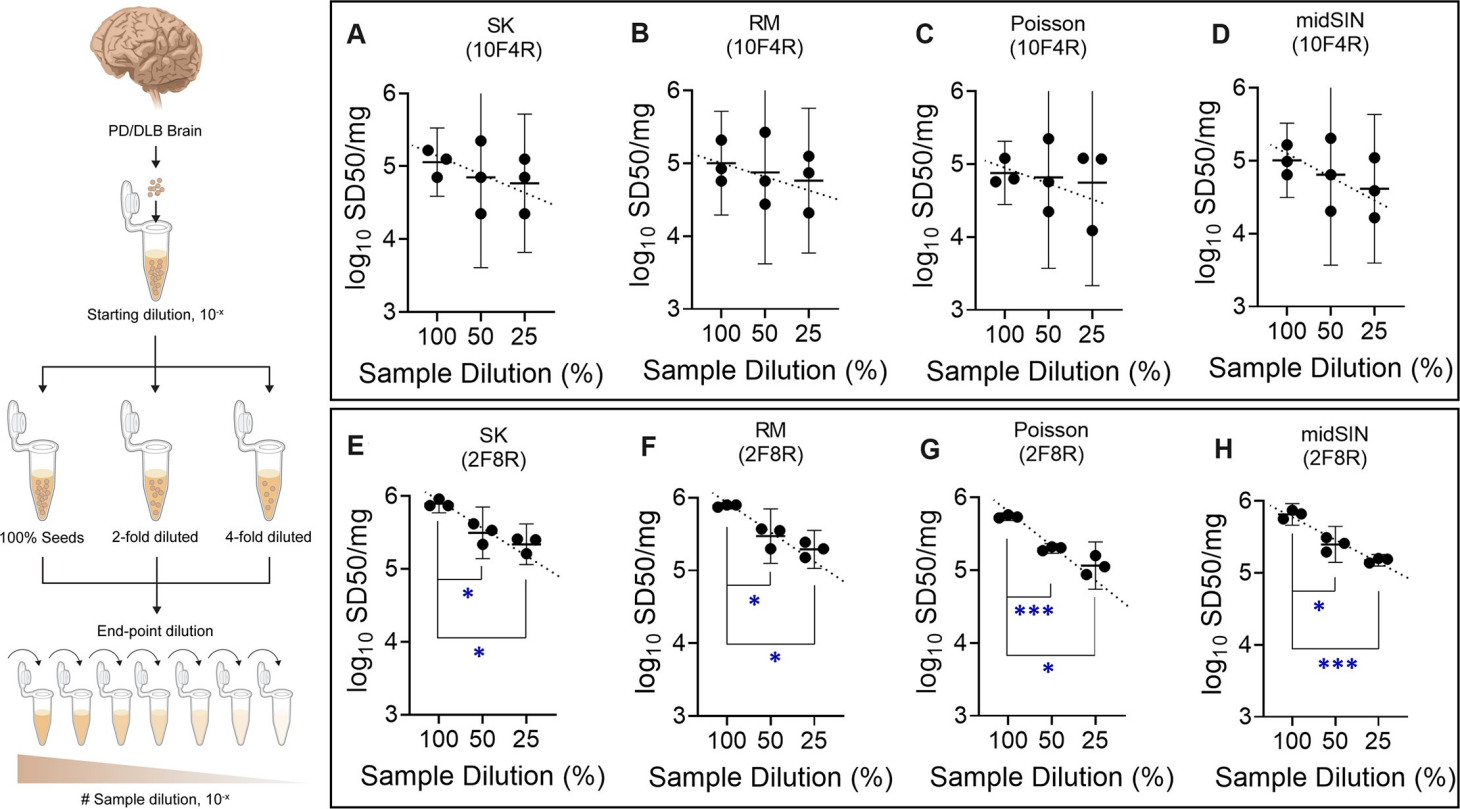
Stratification of samples differing in αSyn^D^ seed concentrations. The schematic on the left shows experimental design for testing 2-fold and 4-fold differences in αSyn^D^ seed concentration in PD brain tissue. Scatter plots showing quantification efficacy of all four algorithms (SK, RM, Poisson and midSIN) in the (A)-(D) 10F4R (top panel) and (E)-(H) 2F8R (bottom panel) ED assay formats. In each plot, log_10_ SD50 values from 3 independent ED assays are displayed (black circles). Error bars represent arithmetic mean with 95% CI. The trend of SD50 estimates in tested sample dilutions are shown as dotted black lines in each plot. Statistical significance is denoted in blue as *p<0.05, **p<0.01 and ***p<0.005.

In the first approach, we generated PD BH samples with 2-fold and 4-fold differences (or log_10_ SD50 differences of 0.3 or 0.6, respectively) in αSyn^D^ by volumetric dilution and applied the four estimation methods to data from both the 10F4R and 2F8R ED assays (Fig 5). In the 10F4R format (Fig 5A–5D, top panel), none of the four estimation methods could distinguish 2-fold or even 4-fold differences in αSyn^D^ seeding activity. This was due primarily to the limited number of wells used overall in the 10F4R ED assay (5 dilutions x 4 replicates = 20 wells). Based on work by Cresta et al [39], SD50 estimation from 20 wells should result in a 95% CI width of about 7-fold (10^±0.55^ SD50/mg). This would preclude statistically significant discrimination of 2-fold or even 4-fold dilutions, especially if other sources of errors added to the measurement’s uncertainty.

In contrast, the 2F8R ED assay (Fig 5E–5H, bottom panel), with its 96 wells (12 dilutions x 8 replicates) and an expected 95% CI width of about 3-fold (10^±0.2^ SD50/mg, based on [39]), could potentially detect 2-fold and 4-fold changes with improved statistical strength. Indeed, in the 2F8R assay, the Poisson and midSIN methods could usually discriminate 2-fold (p<0.01 and p<0.05, respectively) and 4-fold (p<0.05 and p<0.005, respectively) differences in SD50 estimates more precisely than either the SK or RM methods. In both the 10F4R and 2F8R assays, some likely false positive wells were seen at larger dilutions (S5 and S6 Figs). These deviations from theoretical expectations, namely that the percentage of positive wells should decrease monotonically with increasing dilutions, likely contribute further errors to the estimate beyond the accuracy limitations imposed by the number of wells used in performing the ED assay.

### Improved quantitation of αSyn seed reduction in a longitudinal inactivation regime

**Fig 6:**
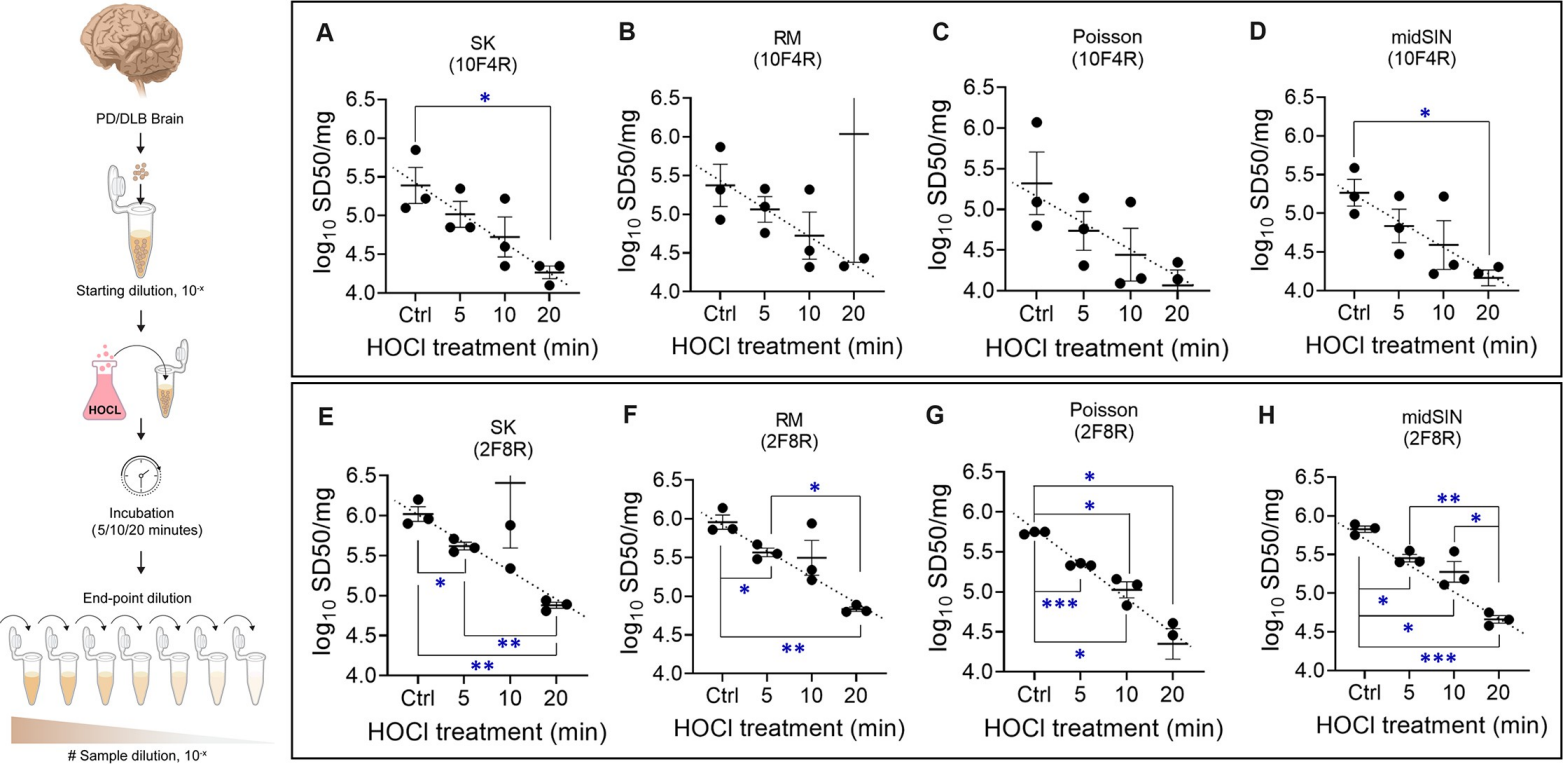
Quantification of time-dependent αSyn^D^ seed reduction by HOCl treatment. The schematic on the left shows experimental design for HOCl treatment of PD BH for different treatment times followed by estimation of differences in αSyn^D^ seed concentration by ED assays. (A)-(H) Scatter plots showing estimate using the SK, RM, Poisson and midSIN algorithms in the 10F4R (A-D) and 2F8R (E-H) ED assay formats. In each plot, log_10_ SD50/mg tissue values from 3 independent ED assays are displayed. In each case, the triplicate ED assay performed on untreated PD BH is shown as control (Ctrl). Error bars represent arithmetic mean with 95% CI. The trend of SD50 estimates with increasing HOCl treatments is shown as dotted black lines in each plot. Statistical significance is denoted in blue as *p<0.05, **p<0.01 and ***p<0.005.

The utility of pathological αSyn^D^ as a pharmacodynamic biomarker for evaluating drug efficacy relies upon the ability of SAAs like RT-QuIC to quantify changes in αSyn^D^ seed load as a function of treatment. To model this capability *in vitro*, we compared samples treated with hypochlorous acid (HOCl), an oxidant that inactivates αSyn^D^ and other pathological amyloid seeds in a time-dependent manner (40). PD BHs treated with 0.1% (v/v) HOCl, for 5, 10, and 20 min were tested using the 10F4R and 2F8R assays. With the 10F4R format, we saw a trend of decreasing SD50 with increasing time of exposure but variability in the SD50 estimates between 3 separate ED RT-QuIC assays compromised the statistical significance of SD50 intervals at different treatment intervals as compared to the untreated control (Figs 6A–6D, top panel and S7). Nonetheless, SK and midSIN based estimations still differentiated samples at the extreme ends of the treatment regime (untreated versus 20 min treatment).

On the other hand, the 2F8R ED assay markedly improved the accuracy of the SD50 estimates (lower 95% CIs) and statistical significance of SD50 differences between different treatment intervals (Figs 6E–6H, bottom panel and S8). Both SK and RM methods differentiated seed load in untreated PD samples from 5 min (p<0.05) and 20 min (p<0.01) HOCl treatments. However, Poisson and midSIN were more robust in their detection of differences in seed loads across all three treatment intervals. In fact, the midSIN estimation discriminated seeding differences between 10 and 20 min (p<0.05) treatments as well as 5 and 20 min (p<0.01) treatments. Importantly, in the 2F8R assay format, the 4 estimation methods generally found a reduction of about 2.6-fold in the SD50 (0.42 in log_10_ SD50) for 5 min treatment with HOCl, 5-fold (0.7 in log_10_ SD50) for 10 min treatment, and 16-fold (1.2 in log_10_ SD50) for 20 min HOCl treatment. No significant difference in αSyn^D^ seeding by untreated and mock treated (milliQ treated) samples was noted (S9 Fig). Overall, these results provided additional evidence that the 2F8R assay enabled discrimination in differences in seed concentration of as little as ~2-fold.

### αSyn seed quantitation in clinical samples from synucleinopathy patients

Ultrasensitive detection of αSyn^D^ in clinically accessible biospecimens from living synucleinopathy patients, such as cerebrospinal fluid (CSF) and peripheral tissues has further boosted the diagnostic utility of SAAs [18,41–43]. So, we next tested *antemortem* CSF, skin, and OM samples from living PD/DLB patients (S1 Table), with the 10F4R, 2F8R, or, in the case of CSF, 2F4R (with 4 replicates per dilution) assays and estimated SD50 concentrations using the different estimation methods. The 2F4R assay format was used for the CSF samples owing to their limited availability.

**Fig 7:**
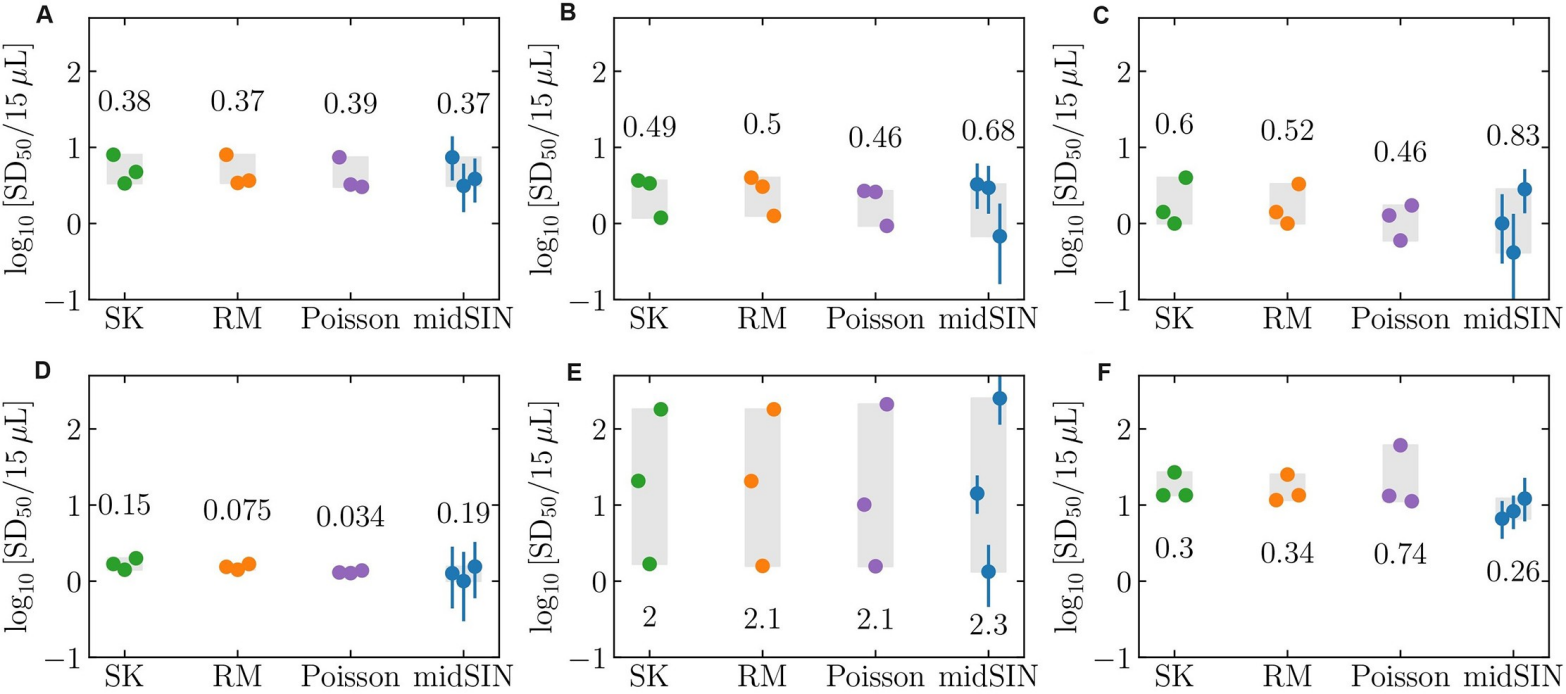
Quantification of αSyn^D^ seeding in patient CSF samples. 2F4R RT-QuIC quantification of αSyn^D^ seeding in patient CSFs. A-F Replicate heterogeneity (grey bars) in log_10_ SD50 estimates for 6 antemortem PD patient CSFs (PD1-PD6) obtained from 3 independent 2F4R ED assays. The log_10_ SD50 values obtained from SK (green), RM (orange), Poisson (violet) and midSIN (blue) 4 methods are shown. The error bars in midSIN estimates represent 95% CI for each replicate based on the amount of noise/confidence of individual SD50 estimates. Each colored circle represents one of the 3 replicates, the numbers correspond to the difference between the minimum and maximum log_10_ SD50/15 μL values obtained, and the shading indicates this [min., max.] range.

The CSF samples were estimated to contain ~1–10 SD50 per 15 μL (Fig 7). In contrast, the BH samples contained 10^4^−10^6^ SD50/mg tissue. Due to their low SD50 concentration, we tested undiluted CSF and dilutions thereof. Variations in the ThT fluorescence curves’ appearance was observed between the undiluted CSF and subsequent dilutions. In many cases, the curves also showed evidence of secondary reactions appearing as sudden changes in the curves’ otherwise regular shape. Some of these sudden changes caused the ThT level to pass the determining threshold for positivity, resulting in large deviation of the ED assay outcome from theoretical expectations (S10 Fig). With the 2F4R assay format, CSF samples can be expected to have a 95% CI width of about 6-fold (10^±0.5^ SD50/mg). Overall, the CSF estimates differed over 3 independent assays by an amount consistent with the expected accuracy, i.e., roughly 0.5 log_10_ SD50/15 μL (PD1-4 & PD6, Fig 7A–7D and 7F). The remaining sample (PD5, Fig 7E) varied more widely between the 3 assays, with one having positive wells at all but the lowest dilution and another having all negative wells for all but the undiluted sample. We cannot rule out technical error in this latter assay but report it here for completeness in case it might truly represent anomalous behavior of aliquots of this CSF specimen.

**Fig 8:**
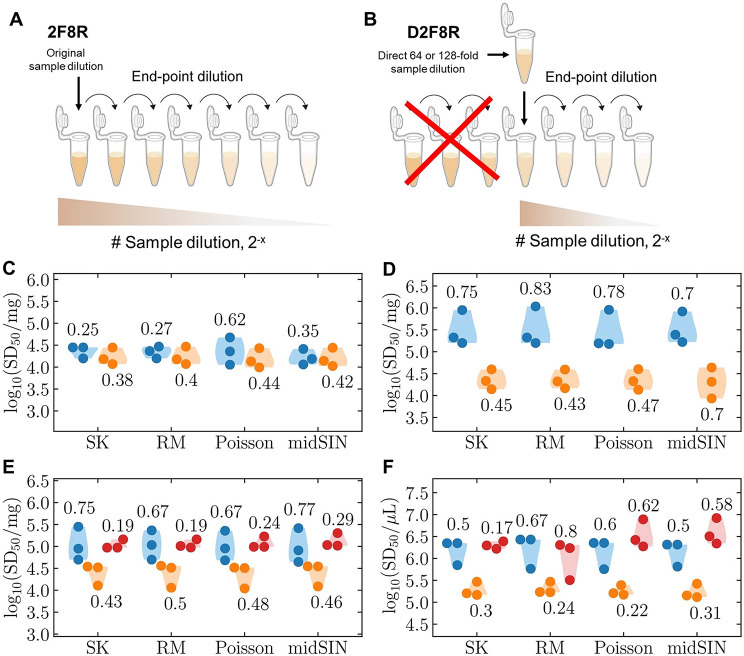
Quantification of αSyn^D^ seeding activity in patient skin and olfactory mucosa (OM) samples. Schematic showing 2F8R (A) and D2F8R (B) assay formats. D2F8R format reduces pipetting steps by directly preparing an intermediate sample dilution to minimize seed loss (see methods). Replicate heterogeneity (colored circles and bars) in log_10_ SD50 estimates for patient skin samples *viz*. (C) PD1, (D) PD2 and (E) DLB 1, and (F) OM sample from a PD patient, obtained from 3 independent 10F4R (blue) and 2F8R (orange) assay formats. Enhanced accuracy of all 4 quantification algorithms (low CIs) was obtained with D2F8R assays (red) as shown with (E) DLB1 skin and (F) PDOM samples. Numerical values above or below each bar represent log_10_ difference between the maximum and minimum SD50 values estimated with a given method between the 3 replicate ED assays. In (C)-(E), the mg denominators in the y-axis labels refer to weight of solid tissue and in (F), the μL denominator refers to volume of packed OM swabbing-derived pellet.

Given that αSyn^D^ seeding activity in both skin and OM of patients has been described as a novel biomarker for the diagnoses of synucleinopathies [10,11,44–46], we applied our 10F4R and 2F8R assays to such specimens. We analyzed skin samples from two PD (Fig 8C and 8D) and one DLB (Fig 8E) patient, each with 3 separate ED assays. The skin samples were estimated to contain 10^4^–10^6^ SD50/mg, i.e., αSyn^D^ levels approaching those in our BH samples. While for the BH samples the 2F8R assay led to more accurate estimates (lower 95% CI) than the 10F4R assay, this effect was marginal in our evaluations of the 3 skin samples (10F4R, blue; 2F8R, orange). This is likely because in the 2F8R assay, the sample’s SD50 was close to the most extreme dilution of the assay: all but 3–5 dilutions out of 12 were 100% positive (S11A–S11F Fig). In the BH samples, where typically 1–3 dilutions out of 12 were 100% positive, we saw how having many such wells can help average over noise from false positive wells at dilutions at or below 1 SD50. In case of skin samples, compared to the 2F8R SD50/mg estimate (orange), the 10F4R estimate (blue) was ~0.5 log_10_ higher for the DLB sample (Fig 8E), and it was either consistent with (Fig 8C) or ~1 log_10_ higher (Fig 8D) for the 2 PD samples. This could either be due to the limitations mentioned above, or to other factors that differ between the 10F4R and 2F8R assay, notably differences in sonication and diluents. We also wondered whether this discrepancy could at least in part be due to a greater loss of seeds to tube walls and pipette tips in the 2F8R format because of the need for several more 2-fold serial dilutions to get to the end-point dilution than was required for the 10-fold dilutions in the 10F4R format when starting from the same initial sample dilution. To address this possibility, we performed additional 2F8R assays (labelled D2F8R, see methods) of one of the same skin samples (Fig 8E, red circles) samples in which 7 of the 2-fold dilutions required were bypassed with a single 128-fold dilution so that the total number of serial dilutions required to get to the initial dilution was comparable to the number required in the 10F4R format. The resulting log_10_ SD50 estimates from the D2F8R assays (Fig 8E, red circles) increased and were more consistent with those determined with the 10F4R format (blue).

Like the BH and skin samples evaluated herein, the OM sample gave estimated concentration of 10^5.2^–10^6.4^ SD50/mg (Figs 8F, S11G, and S11H). As with the skin sample, the SD50 for this OM sample from a PD patient was estimated by the 10F4R assay (orange circles) to be around 6 times that estimated from the 2F8R assay (blue circles). As suggested by the previous experiment with the skin samples, the lower seed load measured by 2F8R is likely due primarily to greater seed losses to tubes and/or pipette tips. We confirmed this by performing additional D2F8R experiment with PDOM sample (starting with single 64-fold dilution) and found more consistent log_10_ SD50 estimates from triplicate ED assays (Fig 8F, red circles).

## Discussion

Improving the accuracy of SAAs in determining relative proteopathic seed concentrations in patients’ biospecimens may improve the value of these assays in diagnoses, prognoses, and the development of therapeutics. Towards this end, we have explored the effects of certain ED RT-QuIC parameters, data analysis algorithms, and sample handling measures on the accuracy and assay-to-assay reproducibility of αSyn^D^ seed concentrations in complex biospecimens from synucleinopathy patients. Our results provide demonstrations of improvements obtained by decreasing the dilution factor, increasing the assay replicate number per dilution, and choosing an optimal algorithm. However, given the diversity of proteopathic seeds, SAAs, and biospecimen types, more targeted assessments of the impacts of these assay parameters will be needed depending on the specific goals of future SAA-based studies.

SD50 estimates were sometimes further aided by more accurate estimation methods (midSIN and Poisson, rather than SK), resulting in >2000-fold higher number of unique assay outcomes (Fig 3). The midSIN and Poisson methods are least affected by the noise in data points resulting from ED assays and so their SD50 estimates can be more accurate and robust than those from conventional SK analysis under some circumstances.

Our 2F8R ED αSyn RT-QuIC assay format discriminated as little as 2-fold differences in endogenous brain seeding activity (Fig 5) and detected a range (~2-16-fold) of seed reductions caused by HOCl treatment (Fig 6). Based on our results so far, we expect that assay accuracy can be further enhanced by using still larger replicate numbers per sample dilution. Given that patients’ biospecimens are often limited, the potential to dramatically increase assay replicate numbers and reduce dilution factors may depend upon miniaturization of SAA assays [32] analogous to what has been accomplished with digital PCR for quantifying nucleic acid in complex tissue samples [47].

Despite the clear indications of the benefits our alternative ED RT-QuIC protocols with synucleinopathy brain specimens, our initial applications of the 2F8R assay format to skin and OM specimens exposed technical issues that should be considered in applications to other types of biospecimens and seeds. With OM and skin, our data suggested that artifactual losses of αSyn^D^ seeds were due to increased numbers of serial sample dilutions in the initial 2F8R assays (Fig 8). This was consistent with previously reported losses of prion seeds and other proteinaceous biomarkers (e.g., Progranulin, Aβ) of neurodegenerative disorders present in biofluids which can adsorb to sample tubes even after short incubations [48–51]. Additionally, changes in measured proteopathic seed concentration (e.g.: αSyn^D^) in biospecimens or dilutions thereof, as measured by ED titration, might also be caused by clumping or fragmentation of seeds during various steps of the ED assay. In any case, alterations in the protocol (D2F8R) to control for such seed losses brought the 10F4R and 2F8R assays into much closer agreement (Fig 8). Both of these sample types (skin and OM) tend to yield less uniform suspensions than brain tissue, which might also help to explain inconsistencies in generating dilution series. For these and other more troublesome biospecimens, it will likely be necessary to improve sample preparation and dilution protocols. We have not typically found CSF to be difficult to handle in this way and were surprised to observe the striking assay-to-assay variability exhibited by one of the six CSF specimens (Fig 7). At this point, we are unable to rule out technical errors in the handling of aliquots of that particular CSF sample. Overall, these results indicate that care must be taken to mitigate and normalize such potential artifactual alterations in seeding activity when comparing panels of a given type of specimen. It will likely be necessary to develop tissue-specific diluents to minimize effects of variations in the (mis)behaviors of various types of seeds and the biospecimen matrices in which they are found.

An additional challenge associated with using CSF as a diagnostic biospecimen is patients’ frequent reluctance to undergo serial lumbar punctures, prompting searches for more accessible tissues/biofluids [11,15,45,52–54]. Our findings with skin and OM samples (Fig 8) support the idea that αSyn^D^ in more accessible biospecimens can be a valuable and quantifiable biomarker. Specifically, skin and OM samples contained SD50 concentrations that are much higher than found in CSF specimens, and comparable to those found in BH samples (10^4^–10^6^ SD50/mg), possibly because peripheral tissues (especially skin) can accumulate αSyn^D^ depositions continuously over months and years, unlike CSF which is replenished every 6–12 h. Nevertheless, the extent to which peripheral αSyn^D^ seed deposits correlate quantitatively with disease duration, severity or various clinical measures requires further investigation [55].

As mentioned above, there are limitations to our current study. Although the quantitative benefits of our alternative ED assay formats are clear when applied to synucleinopathy brain specimens, more thorough analyses, improved handling protocols, and further increases in replicate number per dilution are clearly necessary to fully realize the same benefits using more practical biospecimens. Such important extensions of the current work will likely require larger sample quantities, more complex clinical sample panels, and technical adjustments tailored to specific biospecimen types and study goals.

In conclusion, we have demonstrated how RT-QuIC parameters can improve quantitation of αSyn^D^ seeding activity as a biomarker in the context of synucleinopathies. The basic principles indicated by our findings should also be applicable more broadly to improve quantitation with other types of ED SAA assays such as those being applied to tauopathies [56–58] and prion diseases [59–61]. Improved quantitation should allow more incisive evaluation of pathological seeding activities as quantitative biomarkers for PD, DLB, and other neurodegenerative illnesses, both in terms of prognosis and diagnosis.

## Materials and methods

### Ethics statement

CSF samples from PD patients were collected under protocol #HCB/2017/1004 the Comité Ético de Investigación Clínica del Hospital Clínic de Barcelona. Skin tissues were obtained under ethics protocol # STUDY20181189 from the Institutional Review Board of Case Western Reserve University and University Hospitals. OM samples were obtained from patients with ethical approval under protocol #CE/12/332 from Istituto Superiore di Sanità (Rome). Brain tissue specimens were obtained post-mortem from the Indiana University School of Medicine and the NIH Brain & Tissue repository-California, Human Brain & Spinal Fluid Resource Centre, VA West Los Angeles Medical Center, Los Angeles, California. No additional ethical permission was needed because the samples were taken from deceased, de-identified, consenting individuals. Written informed consent was obtained from patients or their legal representatives for the collection of all specimens (i.e., brain tissue, skin, cerebrospinal fluid, and OM brushings).

### Study design

The primary research question addressed in this study was whether optimizing physical, chemical, and numerical variables could increase the accuracy of ED assays for quantitating αSyn^D^ seeds in patient biospecimens. In a stepwise approach, we evaluated different assay variables using neuropathologically confirmed PD/DLB brain homogenates (BHs). The improved and conventional ED frameworks were then utilized for evaluating the efficacy of four different quantification algorithms *viz*. Spearman-Kärber (SK) [17,29], Reed-Muench (RM) [62], in-house developed Poisson and midSIN [39] methods. We then sought to corroborate our observations with the improved framework in a validation set of diagnostically relevant, live PD and DLB patient biospecimens i.e., CSF, skin and OM. Clinical details about patient biospecimens tested in the study are provided in S1 Table.

### Preparation of brain homogenates

Frozen post-mortem brain tissue samples were obtained from neuropathologically confirmed cases of Parkinson’s disease (PD), dementia with Lewy bodies (DLB), Corticobasal degeneration (CBD), and healthy individuals. Brain homogenates were prepared as 10% (w/v) in ice-cold phosphate-buffered saline (PBS) (pH 7.0) using 1 mm zirconia beads (BioSpec, cat#11079110z) in a Bead Mill 24 (Fisher Scientific). The resulting tissue homogenate were centrifuged at 2,000g for 2 min, supernatants were aliquoted and stored at −80°C until use.

### CSF samples

The samples were obtained through lumbar puncture (LP) in the L3-L4 space using a 22G needle after overnight fasting and in absence of anti-parkinsonian medication. After the LP procedure, CSF visually contaminated with blood was rejected for any further analysis. CSF samples were immediately centrifuged for 10 min at 4,000g and 4°C, aliquoted and stored at −80°C. Pooled and single donor healthy human CSF were purchased from Innovative Research, pre-screened in RT-QuIC, and stored at −80°C until use.

### Skin samples

Samples mainly containing epidermis and dermis were obtained as skin punches (~50–100 mg each). Skin tissues (10% w/v) were pre-treated in a lysis buffer constituting calcium chloride (2 mM) and 0.25% (w/v) collagenase A (Roche) in 1X TBS and incubated at 37°C with mild shaking for 4 hours. Following this, tissue homogenization was carried out in a Bead Mill 24 (Fisher Scientific) for 1 min. The resulting tissue homogenates were centrifuged at 500g for 5 min, supernatants were aliquoted and stored at −80°C until use.

### Olfactory mucosa samples

OM samples were obtained by nasal swabbing from the patients using flocked swabs (FLOQSwabsR; Copangroup, Brescia, Italy), as described previously [63]. Following extraction, swabs were immediately placed into a 5 ml polypropylene tube filled with 0.9% saline. The tubes were then vortexed for 1 min at room temperature in order to separate the cellular material from the swab. Following this, the cell suspension was centrifuged for 20 min at 2000g and 4°C. After removing the supernatant, the remaining OM pellet was frozen at -80°C until analysis. The OM pellets were thawed and centrifuged for 10 minutes at 3220×g at 4°C. A disposable inoculating loop (Fisher) was used to collect approximately 1 to 2 μL of the pellet and transfer it into a tube with 25 μL PBS. The OM pellet suspension was then sonicated until the pellet was dispersed and further diluted for αSyn RT-QuIC analysis.

### Recombinant α-Syn substrate purification

Recombinant K23Q αSyn substrate purification was carried out as described previously [9]. Briefly, freshly transformed *E*. *coli* cells were pre-screened for protein expression followed by overnight growth as flask culture in auto-induction media at 37°C, 225 rpm [64]. Next day, osmotic shock based periplasmic extraction was performed followed by enrichment of αSyn recombinant protein by acid precipitation. The protein extract was purified using a two-step chromatographic separation (Ni-NTA followed by anion exchange) on an Äkta Pure chromatography system (GE). The resulting protein peak fractions were combined and extensively dialyzed against milliQ water using a 3 kDa MWCO membrane. Dialyzed protein was 0.22 μM filtered and the concentration was determined using a NanoDrop 2000 spectrophotometer and a molecular extinction coefficient (ε_280_) of 5960 M^-1^ cm^-1^. The protein samples were aliquoted (1 mg/ml), lyophilized and stored at -80°C until further use.

### ED assay implemented in αSyn RT-QuIC

ED assay implemented in the RT-QuIC assay was performed using black, clear bottom 96-well plates (Nalgene Nunc International) preloaded with 6 silica beads (0.8 mm diameter, OPS Diagnostics). Designated wells were supplemented with 98 μL of reaction buffer (40 mM phosphate buffer, pH 8.0 and 170 mM NaCl) containing 0.1 mg/ml αSyn K23Q substrate (prefiltered through 100 kDa MWCO filter, Pall Corporation, Catalog # OD100C34) and 10 μM ThT. Replicate wells (n = 4) were seeded by 2 μL of subsequent 10-fold BH dilutions (10F4R) made in PBS and the plate was covered with a sealer film (Nalgene Nunc International) and incubated at 42°C in a fluorescent plate reader (BMG FLUOstar Omega) with 1 min shake-rest cycles (400 rpm double orbital) for at least 50 h. Fluorescence reads were taken every 45 min using 450–10 nm excitation and 480–10 nm emission filters. For both skin and OM samples, RT-QuIC assays were run with the above-mentioned protocol using 2 μL (or 2 mg) of the samples.

For 2F8R assay, brain, skin homogenates and OM samples were pre-processed via a pre-sonication step (20 kHz, power setting: 1, 3 s burst, 10 cycles) followed by serial 2-fold dilutions in a diluent constituting PBS/N2 with 0.0001% negative biospecimen (CBD) and seeding replicate wells (n = 8) per dilution. D2F8R assay format involved direct dilution of samples to an intermediate dilution (64-fold; OM or 128-fold; skin), followed by ED to reduce 50% of the total number of dilutions (or pipetting intervals) to minimize seed loss. In case of CSF samples, 2-fold serial dilutions were made in healthy/normal CSFs and 15 μL of subsequent dilutions were seeded in 85 μL of reaction buffer per well. Here, the reaction buffer included 0.0015% sodium dodecyl sulfate (SDS) supplemented with other constituents as described above. ED assay was performed with 4 replicates per dilutions (2F4R) for optimal assay design in a 96-well assay format.

### Positive well determination

For the conventional assay, the ThT fluorescence curve in each well of the assay was first normalized by dividing it by the geometric mean of all ThT fluorescence measurements taken in that well between 2 h and 7 h. A well was considered positive if its normalized ThT fluorescence increased to at least 2.8 times its baseline by or before 50 h. In the modified assay format, a fluorescence growth rate threshold of 0.01/h was used but was evaluated only at 40 h such that a well was considered positive if it met the following criteria expressed in Eq 1:

$$Growth\;rate=\frac{\left[log_{10}ThT(40\;h)-log_{10}ThT(5\;h)\right]}{\left(40h-5h\right)}>0.01/\text{h}$$ (1)

where ThT (40 h) and ThT (5 h) are the ThT fluorescence measured at 5 h and 40 h, respectively, and 0.01/h is our chosen growth rate threshold. This growth rate-based threshold does not require that the ThT fluorescence be normalized and is easily adapted to a different time cut-off (e.g., 50 h or even 24 h). In fact, the 2.8-fold threshold adopted here for the conventional assay also corresponds to a growth rate of 0.01/h over 50 h, and to that extent these two different ways of defining a positive well are mathematically equivalent.

### Alternate SD50 estimation methods

The RM (Reed-Muench) method is most closely related to the SK method. It differs from the latter by first transforming the data, including taking the cumulative sum of positive and negative wells, which ensures that the fraction of positive well decreases monotonically with increasing dilution, and then estimates the SD50 from the transformed data. Like the SK method, it is most reliable when the SD50 is at or near the middle dilution in the series but performs poorly if the sample dilution estimated to contain 1 SD50 lies near the lowest or highest dilution in the assay [62]. Because it relies on approximations similar to those of the SK method, it also systematically overestimates the SD50 by 23% [35,39].

The Poisson model (Poisson) assumes that the number of positive wells at each dilution follows a Poisson distribution in which the predicted rate of positive wells at each dilution depends on the number of seeds in the sample. The SD50 concentration is then estimated by minimizing the sum of squares of errors between the data and the Poisson’s predictions, assuming equal weight on all points. It is more robust and accurate than the SK and RM methods because it uses data from all, rather than a subset, of the sample dilutions in the series, and relies on the actual underlying function (Poisson distribution) rather than an approximation of it; hence, the Poisson avoids the overestimation of the SK and RM methods.

The midSIN estimation method was originally developed to quantify the concentration of infectious virus in samples and relies on Bayesian inference to estimate the sample dilution giving 50% positive responses in an ED assay [39]. One of the major advantages of midSIN is that it successively revises and improves SD50 estimates based on information obtained at each dilution in the ED assay. As a result, midSIN’s accuracy is increased as it can account for even large deviations from theoretically expected ED assay outcomes. One key difference between the Poisson and midSIN estimator is that the Poisson assigns the same weight to all points in fitting the fraction of seed-positive wells at all dilutions whereas midSIN assigns lower weight to more extreme dilutions, where the lower sample concentration yields more variable and thus less reliable outcomes.

### Simulated ED RT-QuIC experiments

Simulated ED assays assume that the probability that a well becomes positive will depend on the SD50 concentration of the neat sample (C_sample_), the total volume of sample + diluent added to each assay well (V_well_), and the specific dilution of the sample for that well (Dil), given by Eq 2:

$${\text{P}}_{\text{pos}}=1-\text{exp}\left[{-\text{C}}_{\text{sample}}{\times \text{V}}_{\text{well}}\times \text{Dil}\right]$$ (2)

For example, if a total volume of V_well_ = 2 μL is added to each ED well, and the sample has a concentration of C_sample_ = 10^4^ SD50/μL, in an ED assay column where the sample is diluted by 10,000-fold (Dil = 0.0001), the probability that a well in this column becomes positive is 86.5%. In the simulated ED assay, the random number of positive well out of a total of N_rep_ replicate wells at each dilution (where N_rep_ is either 4 or 12 replicates) is then drawn at random from a binomial distribution, based on n = N_reps_ draws and a likelihood of success, p = P_pos_. This process is repeated for each column of the assay, where the dilution (Dil), and therefore P_pos, changes between the different columns of the assay, yielding a random number of positive well for each column, e.g. [4/4, 3/4, 0/4, 0/4, etc.], i.e. the outcome of one randomly generated plate. This process is repeated one million times, potentially resulting in several duplicates of any randomly generated plate outcome, and the estimated SD50 concentration of each plate outcome is estimated by each of the 4 methods explored: SK, RM, Poisson, and midSIN.

### Estimation of the SD50 from the ED assay outcome

Estimation of the SD50 following the Spearman-Karber method [17,29], the Reed-Muench method [62], or the midSIN method [39] was performed as described in the papers that originally introduced these methods. What is referred to herein as the Poisson model which is based on the following function [Eq 3]:

$${\text{theo}}_{\text{pos}}(x)=1-\text{exp}\left[\frac{{-\text{V}}_{\text{inoc}}\times \text{SD50}\times x}{\text{ln(2)}}\right]$$ (3)

where theo_pos_ is the fraction of positive wells theoretically expected at a given dilution ***x*** (e.g., a 1:100 sample dilution would correspond to ***x*** = 0.01) given an inoculated sample volume (V_inoc_) placed in each well of the assay. The SD50 is estimated by seeking its value such that it will minimize the sum of squared residuals between the theoretically expected fraction of positive wells, theo_pos_(***x***), and the experimentally observed fraction of seeded wells, obs_pos_(***x***), namely [theo_pos_(***x***)–obs_pos_(***x***)]^2^ summed over all dilutions ***x***. The python function scipy. Optimize.curve_fit was used to fit the theoretical curve to the observations to estimate the SD50. Standard error (SE) in SD50 estimations by SK method was calculated as described previously [65] using the following Eq 4:

$$SE=\sqrt{d^{2}\sum \frac{p(1-p)}{n-1}}$$ (4)

Where, *p* = positive well proportion at a given dilution, *n* = number of replicates and *d* = log_10_ (dilution factor).

### HOCl treatments and ED assay

10% PD BH (w/v) was incubated at room temperature for 0, 5, 10 and 20 minutes after mixing with a 100-fold (v/v) excess of a 1% dilution of BrioHOCl stock solution (BrioTech, Inc.). Following incubation, 10- and 2-fold serial dilutions were prepared as described above and ED was carried out using the αSyn RT-QuIC assay. ED outcomes were analyzed using the four quantification algorithms for quantitating seeding dose producing positive outcomes in 50% of the replicates wells tested for each dilution (SD50).

### Statistical analysis

Statistical comparisons were performed using t-tests and one-way ANOVA for comparing two or more groups, respectively in GraphPad Prism Software Version 9.0.0. Data were expressed as scatter plots showing mean and 95% CI (confidence intervals). Here, the lower and upper confidence limits were computed as arithmetic mean ± 4.303 SEM. A two-sided type I error level of 0.05 was adopted. The statistically significant differences were expressed as * p<0.05, ** p<0.01, *** p<0.005, and **** p<0.001 as indicated.

## Supporting information

S1 Fig Primary ThT fluorescence data of αSyn ED RT-QuIC analyses with varying dilution factors. Panels show traces from 4 replicate reactions at the designated dilutions (shown on top of each graph) resulting from serially 10-, 5- and 2-fold diluted PD BHs. The fractions in the left corner of each graph indicate the number of ThT-positive wells at each dilution.
(DOCX)

S2 Fig Reduction in standard error in log_10_ SD50 estimates as a function of dilution interval and replicate number. (A), (C) Standard error (SE) in calculating log_10_ SD50/mg for 10-, 5- and 2-fold dilution series in ED RT-QuIC assays performed for PD and DLB BHs, respectively. (B), (D) Comparative plot showing SE in log_10_ SD50/mg for 10-, and 2-fold dilution series for 4, 8 and 12 replicates in ED assays performed for PD and DLB BHs, respectively. SE in SK-based log_10_ SD50 estimates was calculated as described earlier (see Methods). In each case, colored circles represent SE from triplicate ED assays with horizontal bar showing the mean value.
(DOCX)

S3 Fig Comparison of different replicate numbers in RT-QuIC assays using a 2-fold dilution series for PD (A-B) and DLB (C-D) BHs. (A), (C) Percentage of positive wells from 3 independent RT-QuIC assays performed for different number of replicates per dilution (4, 8 and 12 replicates; colored circles) as a function of the dilution (2-folds). (B), (D) Scatter plot of the SK-estimated log_10_ SD50 values for 3 independent ED assays as a function of the number of replicates per dilution represented as arithmetic mean (horizontal bars) and 95% CIs (vertical bars).
(DOCX)

S4 Fig Example outcomes for (A, B) 10F4R and (C, D) 2F8R assays in RT-QuIC ED experiments. (A), (C) Observed number of positive wells in the RT-QuIC assay (black circles) and expected number of wells with seeds as a function of sample dilution for the most likely value of the log_10_ SD50/mg estimated via the SK (green solid), RM (orange dashed), Poisson (purple dotted), or midSIN (blue dash-dot) methods (indicated in this order above the graph); the short vertical lines indicate the sample dilution at which ED wells would be expected to receive 1 SD50. (B), (D) Posterior likelihood distribution for the log_10_ SD50/mg estimated by midSIN, with the vertical lines corresponding to estimates for each method.
(DOCX)

S5 Fig Assessment of 10F4R ED assay for differentiating 2-and 4-fold dilutions of PD BH. Outcomes from three independent ED assays performed separately for all 3 sample dilutions viz. (A)-(C) neat (100%), (D)-(F) 2-fold diluted, and (G)-(I) 4-fold diluted BH are displayed. The graphs are as described in the caption of S4 Fig (A,C).
(DOCX)

S6 Fig Assessment of 2F8R ED assay for differentiating 2-and 4-fold dilutions of PD BH. Outcomes from three independent ED assays performed separately for all 3 sample dilutions viz. (A)-(C) neat (100%), (D)-(F) 2-fold diluted, and (G)-(I) 4-fold diluted BH are displayed. The graphs are as described in the caption of S4 Fig (A,C).
(DOCX)

S7 Fig Assessment of 10F4R ED assay for differentiating αSyn^D^ seeding for different durations of HOCl treatment. Outcomes from three independent ED assays performed separately for all 4 HOCl treatment types viz. (A)-(C) Untreated, (D)-(F) 5 min treated, (G)-(I) 10 min treated and, (J)-(L) 20 min treated PD BH are displayed. The graphs are as described in the caption of S4 Fig (A,C).
(DOCX)

S8 Fig Assessment of 2F8R ED assay for differentiating αSyn^D^ seeding for different durations of HOCl treatment. Outcomes from three independent ED assays performed separately for all 4 HOCl treatment types viz. (A)-(C) Untreated, (D)-(F) 5 min treated, (G)-(I) 10 min treated and, (J)-(L) 20 min treated PD BH are displayed. The graphs are as described in the caption of S4 Fig (A,C).
(DOCX)

S9 Fig Comparison of untreated and mock treated PD BHs. Heterogeneity in log_10_ SD50 values obtained from independent 2F8R RT-QuIC ED assays performed in triplicate (black circles) for untreated control (Ctrl) and milliQ (H_2_O) treated PD BHs. The arithmetic mean and 95% CI are shown. Lack of statistical significance is denoted by ‘ns’ (p values > 0.05).
(DOCX)

S10 Fig Variability of outcomes and noise in CSF ED assays. A(i)-C(i) Outcomes from three independent 2F4R ED assays performed separately for CSF dilutions (PD6) are shown. Black arrows show ThT outcome at two different dilutions (broken red circles, 1/2^2^ and1/2^5^) tested in 3 independent ED assays. A(ii)-(iii), B(ii)-(iii), and C(ii)-(iii) show ThT outcomes (colored traces) for individual quadruplet wells for the tested CSF dilution (ii, 1/2^2^; iii, 1/2^5^ in each case). Healthy CSFs tested in quadruplicate wells were used as controls. A(iv)-(v), B(iv)-(v), and C(iv)-(v) show example ThT outcomes for quadruplet wells for tested healthy CSFs. In each case, ThT threshold (horizontal broken line) and time cut-off (vertical broken line) for deciding positive/negative wells are shown.
(DOCX)

S11 Fig Assessment of 10F4R and 2F8R ED assays in quantifying αSyn^D^ seeding skin and OM samples from PD and DLB patients. Outcomes from three independent ED assays performed on skin samples from (A)-(D) two PD patients; (E)-(F) one DLB patient; and (G)-(H) one OM sample from a PD patient, each performed using either the 10F4R (A, C, E, G) or 2F8R (B, D, F, H) assay layout. The graphs are as described in the caption of S4 Fig (A,C). The mg denominators in (A)-(F) refer to weight of solid tissue and the μL denominators in (G)-(H) refer to volume of packed OM swabbing-derived pellet.
(DOCX)

S1 Table Table showing clinical and neuropathological characteristics of synucleinopathy and non-synucleinopathy CSF, skin and OM samples utilized in the present study. (DOCX)

S1 Appendix Excel file with primary data used to assemble figures. (XLSX)
