# Supplementary material for: Enhanced quantitation of pathological α-synuclein in patient biospecimens by RT-QuIC seed amplification assays
Source: PLoS Pathog. 2024 Sep 20;20(9):e1012554. doi: 10.1371/journal.ppat.1012554 (PMC11451978; doi:10.1371/journal.ppat.1012554)
Supplement: S1 Fig — Panels show traces from 4 replicate reactions at the designated dilutions (shown on top of each graph) resulting from serially 10-, 5- and 2-fold diluted PD BHs. The fractions in the left corner of each graph indicate the number of ThT-positive wells at each dilution. (DOCX) [file ppat.1012554.s001.docx]

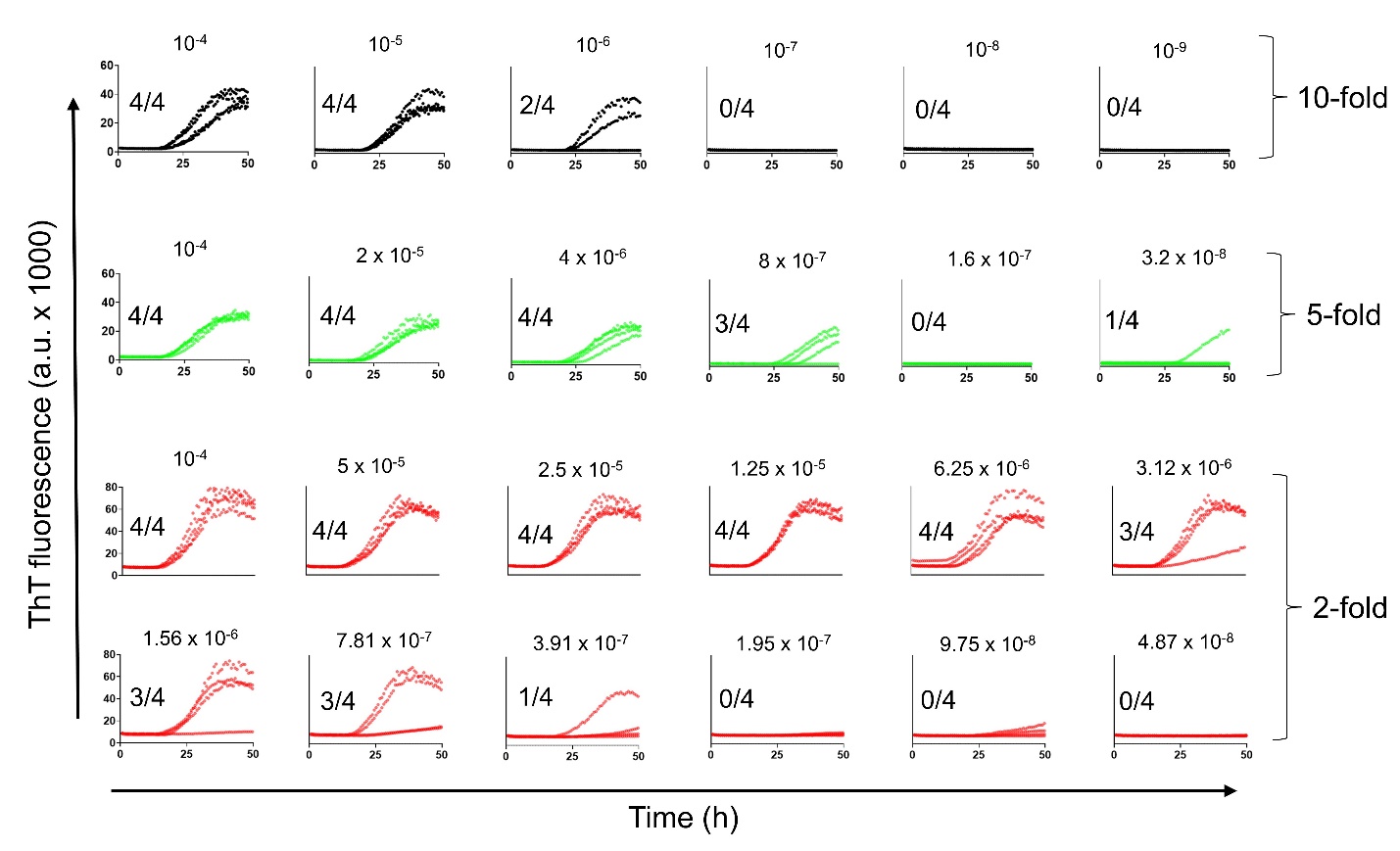


**S1 Fig.** Primary ThT fluorescence data of αSyn RT-QuIC ED analyses with varying dilution factors. Panels show traces from 4 replicate reactions at the designated dilutions (shown on top of each graph) resulting from serially 10-, 5- and 2-fold diluted PD BHs. The fractions in the left corner of each graph indicate the number of ThT-positive wells at each dilution.
