# Supplementary material for: Enhanced quantitation of pathological α-synuclein in patient biospecimens by RT-QuIC seed amplification assays
Source: PLoS Pathog. 2024 Sep 20;20(9):e1012554. doi: 10.1371/journal.ppat.1012554 (PMC11451978; doi:10.1371/journal.ppat.1012554)
Supplement: S2 Fig — (A), (C) Standard error (SE) in calculating log10 SD50/mg for 10-, 5- and 2-fold dilution series in ED RT-QuIC assays performed for PD and DLB BHs, respectively. (B), (D) Comparative plot showing SE in log10 SD50/mg for 10-, and 2-fold dilution series for 4, 8 and 12 replicates in ED assays performed for PD and DLB BHs, respectively. SE in SK-based log10 SD50 estimates was calculated as described earlier (see Methods). In each case, colored circles represent SE from triplicate ED assays with horizontal bar showing the mean value. (DOCX) [file ppat.1012554.s002.docx]

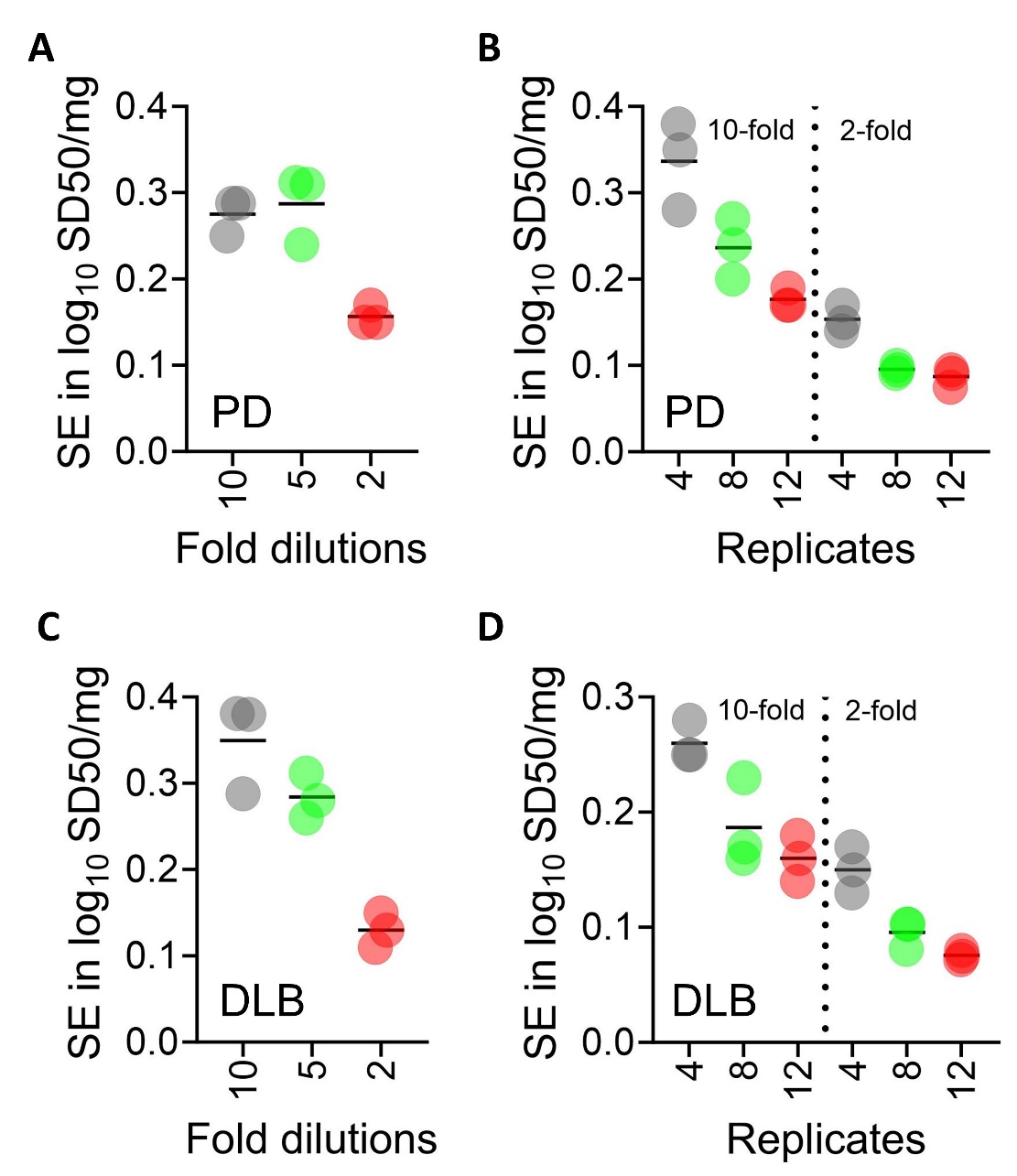


**S2 Fig.** Reduction in standard error in log_10_ SD50 estimates as a function of dilution interval and replicate number. (A), (C) Standard error (SE) in calculating log_10_ SD50/mg for 10-, 5- and 2-fold dilution series in ED RT-QuIC assays performed for PD and DLB BHs, respectively. (B), (D) Comparative plot showing SE in log_10_ SD50/mg for 10-, and 2-fold dilution series for 4, 8 and 12 replicates in ED assays performed for PD and DLB BHs, respectively. SE in SK-based log_10_ SD50 estimates was calculated as described earlier (see Methods). In each case, colored circles represent SE from triplicate ED assays with horizontal bar showing the mean value.
