# Supplementary material for: Enhanced quantitation of pathological α-synuclein in patient biospecimens by RT-QuIC seed amplification assays
Source: PLoS Pathog. 2024 Sep 20;20(9):e1012554. doi: 10.1371/journal.ppat.1012554 (PMC11451978; doi:10.1371/journal.ppat.1012554)
Supplement: S3 Fig — (A), (C) Percentage of positive wells from 3 independent RT-QuIC assays performed for different number of replicates per dilution (4, 8 and 12 replicates; colored circles) as a function of the dilution (2-folds). (B), (D) Scatter plot of the SK-estimated log10 SD50 values for 3 independent ED assays as a function of the number of replicates per dilution represented as arithmetic mean (horizontal bars) and 95% CIs (vertical bars). (DOCX) [file ppat.1012554.s003.docx]

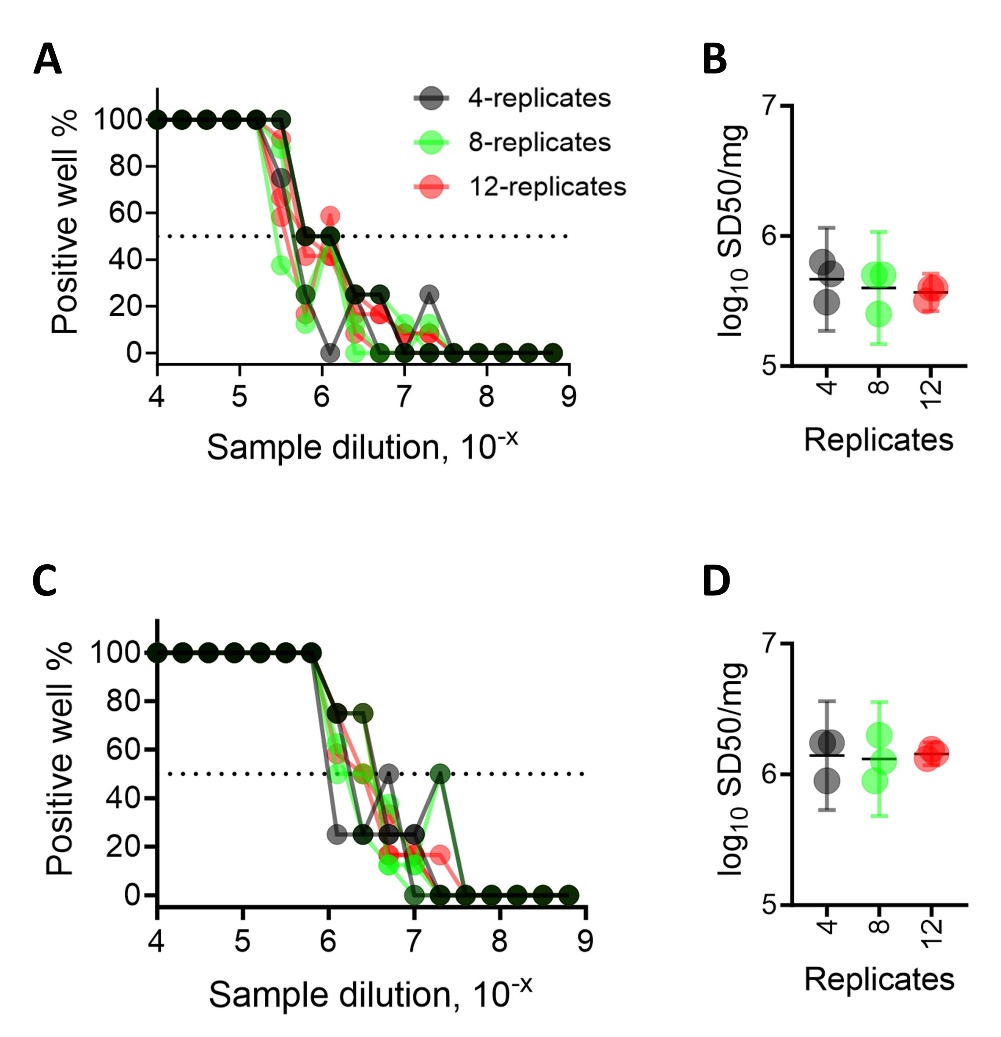


**S3 Fig.** Comparison of different replicate numbers in RT-QuIC assays using a 2-fold dilution series for PD (A-B) and DLB (C-D) BHs. (A), (C) Percentage of positive wells from 3 independent RT-QuIC assays performed for different number of replicates per dilution (4, 8 and 12 replicates; colored circles) as a function of the dilution (2-fold). (B), (D) Scatter plot of the SK-estimated log_10_ SD50 values for 3 independent ED assays as a function of the number of replicates per dilution represented as arithmetic mean (horizontal bars) and 95% CIs (vertical bars).
