# Supplementary material for: Enhanced quantitation of pathological α-synuclein in patient biospecimens by RT-QuIC seed amplification assays
Source: PLoS Pathog. 2024 Sep 20;20(9):e1012554. doi: 10.1371/journal.ppat.1012554 (PMC11451978; doi:10.1371/journal.ppat.1012554)
Supplement: S4 Fig — (A), (C) Observed number of positive wells in the RT-QuIC assay (black circles) and expected number of wells with seeds as a function of sample dilution for the most likely value of the log10 SD50/mg estimated via the SK (green solid), RM (orange dashed), Poisson (purple dotted), or midSIN (blue dash-dot) methods (indicated in this order above the graph); the short vertical lines indicate the sample dilution at which ED wells would be expected to receive 1 SD50. (B), (D) Posterior likelihood distribution for the log10 SD50/mg estimated by midSIN, with the vertical lines corresponding to estimates for each method. (DOCX) [file ppat.1012554.s004.docx]

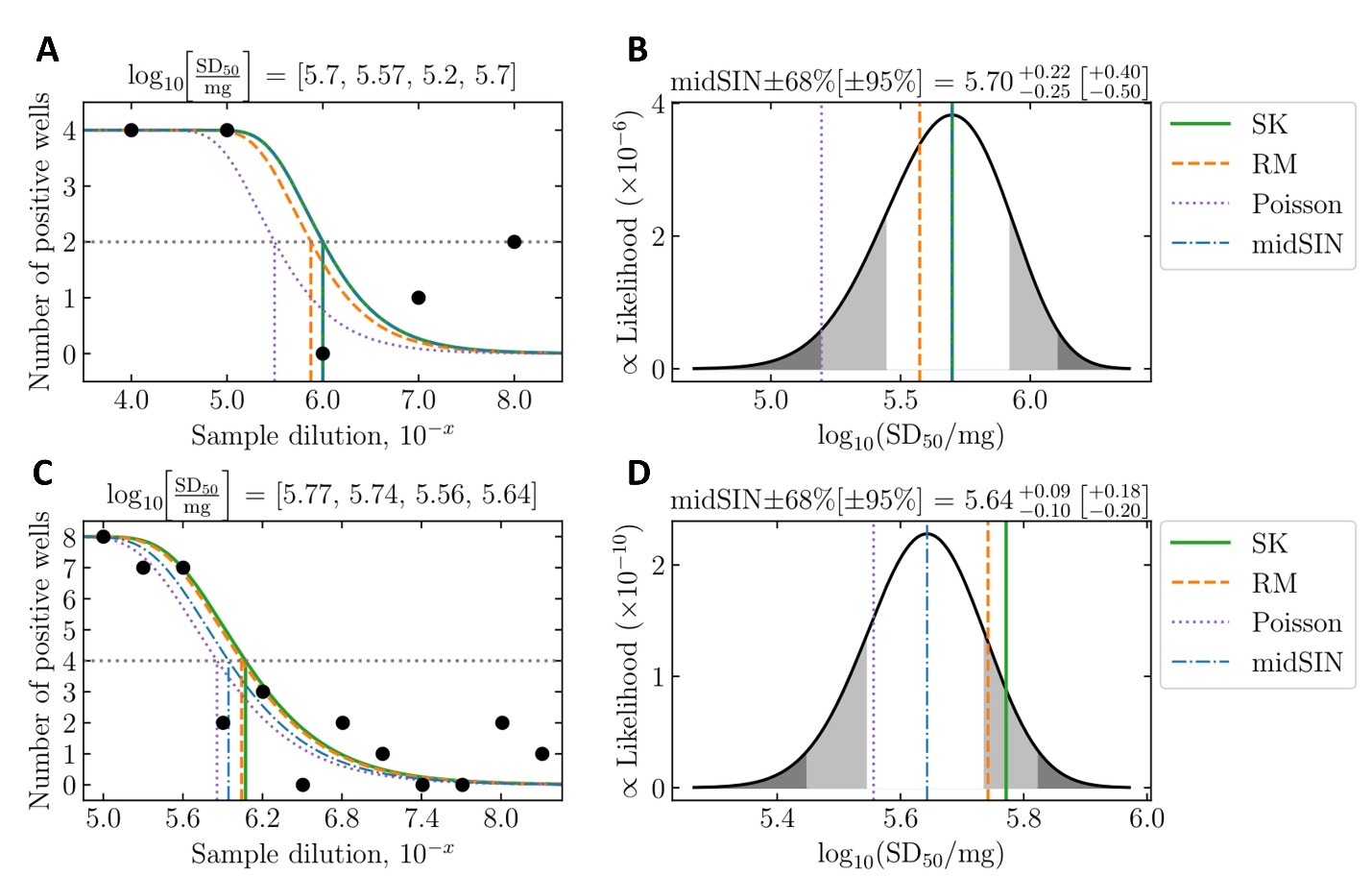


**S4 Fig.** Example outcomes for (A, B) 10F4R and (C, D) 2F8R assays in RT-QuIC ED experiments. (A), (C) Observed number of positive wells in the RT-QuIC assay (black circles) and expected number of wells with seeds as a function of sample dilution for the most likely value of the log_10_ SD50/mg estimated via the SK (green solid), RM (orange dashed), Poisson (purple dotted), or midSIN (blue dash-dot) methods (indicated in this order above the graph); the short vertical lines indicate the sample dilution at which ED wells would be expected to receive 1 SD50. (B), (D) Posterior likelihood distribution for the log_10_ SD50/mg estimated by midSIN, with the vertical lines corresponding to estimates for each method.
