# Supplementary material for: Enhanced quantitation of pathological α-synuclein in patient biospecimens by RT-QuIC seed amplification assays
Source: PLoS Pathog. 2024 Sep 20;20(9):e1012554. doi: 10.1371/journal.ppat.1012554 (PMC11451978; doi:10.1371/journal.ppat.1012554)
Supplement: S5 Fig — Outcomes from three independent ED assays performed separately for all 3 sample dilutions viz. (A)-(C) neat (100%), (D)-(F) 2-fold diluted, and (G)-(I) 4-fold diluted BH are displayed. The graphs are as described in the caption of S4 Fig (A,C). (DOCX) [file ppat.1012554.s005.docx]

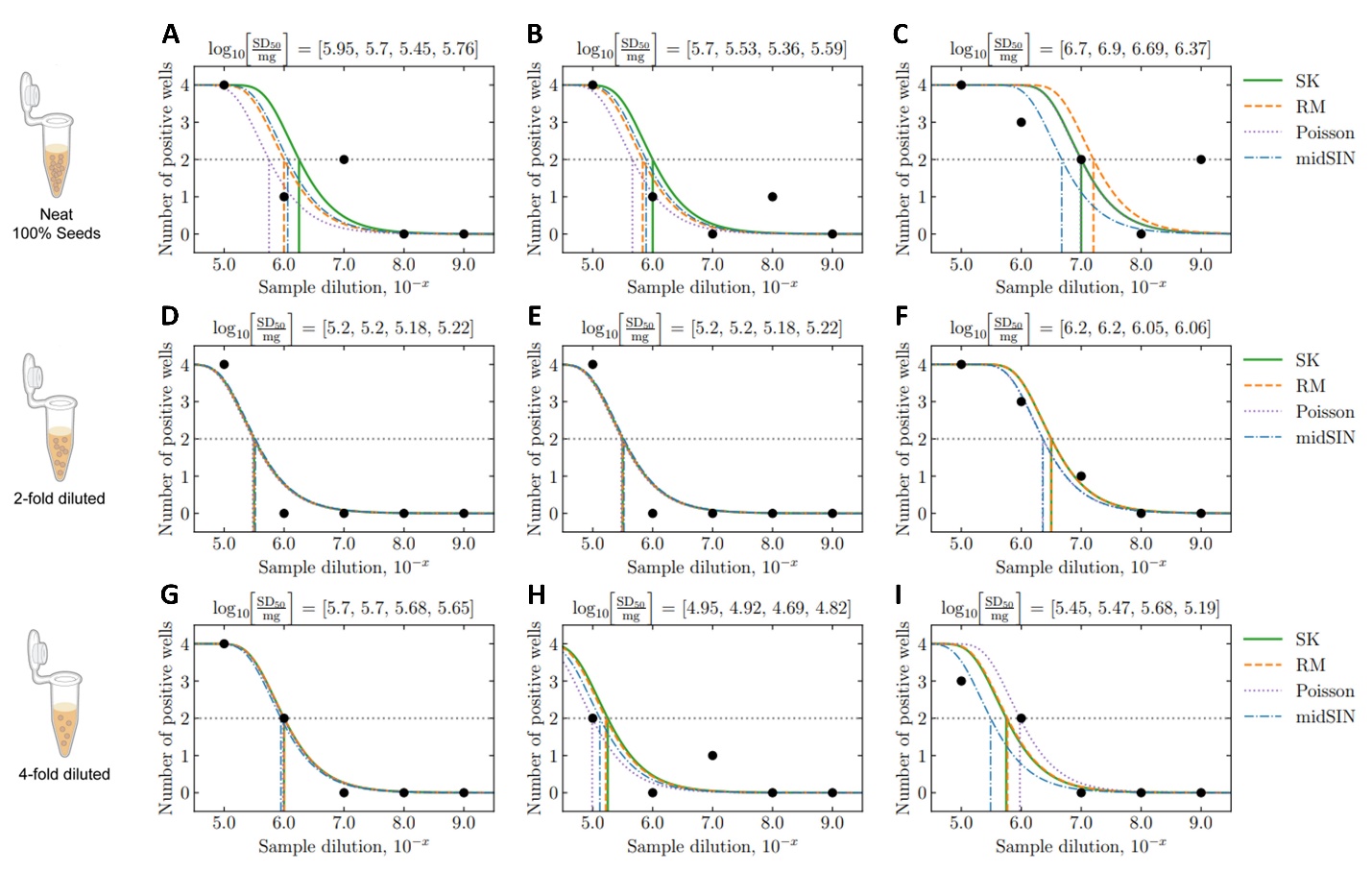


**S5 Fig.** Assessment of 10F4R ED assay for differentiating 2-and 4-fold dilutions of PD BH. Outcomes from three independent ED assays performed separately for all 3 sample dilutions *viz*. (A)-(C) neat (100%), (D)-(F) 2-fold diluted, and (G)-(I) 4-fold diluted BH are displayed. The graphs are as described in the caption of S4 Fig (A,C).
