# Supplementary material for: Enhanced quantitation of pathological α-synuclein in patient biospecimens by RT-QuIC seed amplification assays
Source: PLoS Pathog. 2024 Sep 20;20(9):e1012554. doi: 10.1371/journal.ppat.1012554 (PMC11451978; doi:10.1371/journal.ppat.1012554)
Supplement: S7 Fig — Outcomes from three independent ED assays performed separately for all 4 HOCl treatment types viz. (A)-(C) Untreated, (D)-(F) 5 min treated, (G)-(I) 10 min treated and, (J)-(L) 20 min treated PD BH are displayed. The graphs are as described in the caption of S4 Fig (A,C). (DOCX) [file ppat.1012554.s007.docx]

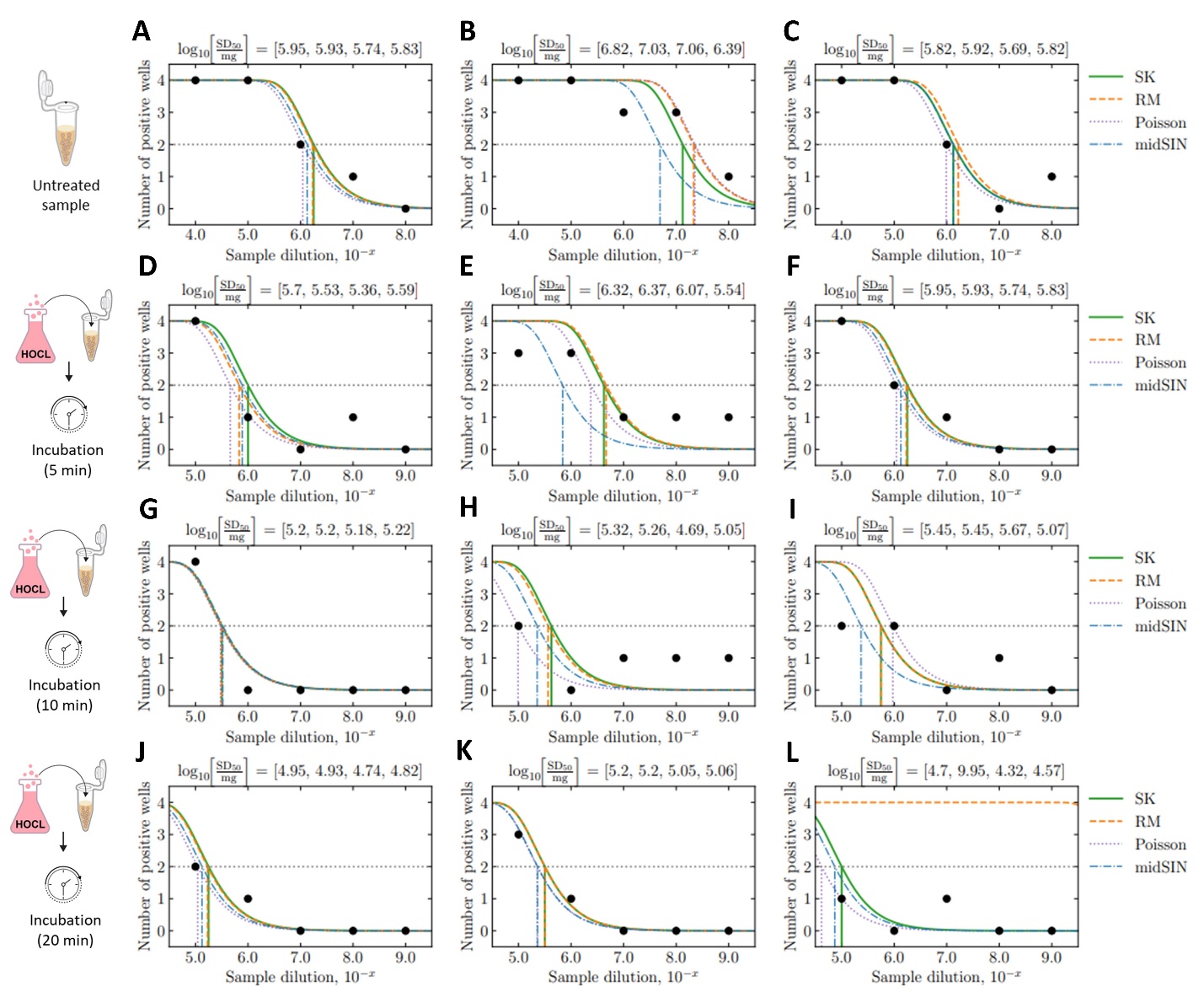


**S7 Fig.** Assessment of 10F4R ED assay for differentiating αSyn^D^ seeding for different durations of HOCl treatment. Outcomes from three independent ED assays performed separately for all 4 HOCl treatment types *viz*. (A)-(C) Untreated, (D)-(F) 5 min treated, (G)-(I) 10 min treated and, (J)-(L) 20 min treated PD BH are displayed. The graphs are as described in the caption of S4 Fig (A,C).
