# Supplementary material for: Enhanced quantitation of pathological α-synuclein in patient biospecimens by RT-QuIC seed amplification assays
Source: PLoS Pathog. 2024 Sep 20;20(9):e1012554. doi: 10.1371/journal.ppat.1012554 (PMC11451978; doi:10.1371/journal.ppat.1012554)
Supplement: S9 Fig — Heterogeneity in log10 SD50 values obtained from independent 2F8R RT-QuIC ED assays performed in triplicate (black circles) for untreated control (Ctrl) and milliQ (H2O) treated PD BHs. The arithmetic mean and 95% CI are shown. Lack of statistical significance is denoted by ‘ns’ (p values > 0.05). (DOCX) [file ppat.1012554.s009.docx]

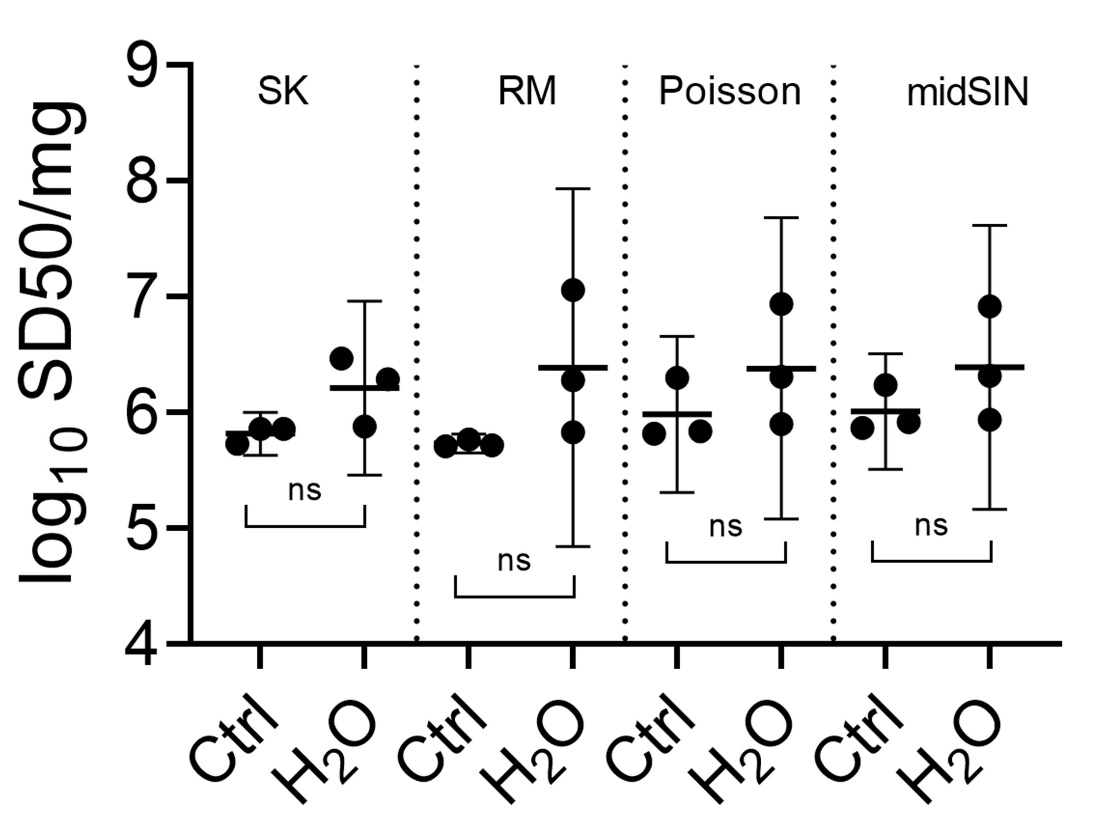


**S9 Fig.** Comparison of untreated and mock treated PD BHs. Heterogeneity in log_10_ SD50 values obtained from independent 2F8R RT-QuIC ED assays performed in triplicate (black circles) for untreated control (Ctrl) and milliQ (H_2_O) treated PD BHs. The arithmetic mean and 95% CI are shown. Lack of statistical significance is denoted by ‘ns’ (p values > 0.05).
