# Supplementary material for: Enhanced quantitation of pathological α-synuclein in patient biospecimens by RT-QuIC seed amplification assays
Source: PLoS Pathog. 2024 Sep 20;20(9):e1012554. doi: 10.1371/journal.ppat.1012554 (PMC11451978; doi:10.1371/journal.ppat.1012554)
Supplement: S10 Fig — A(i)-C(i) Outcomes from three independent 2F4R ED assays performed separately for CSF dilutions (PD6) are shown. Black arrows show ThT outcome at two different dilutions (broken red circles, 1/22 and1/25) tested in 3 independent ED assays. A(ii)-(iii), B(ii)-(iii), and C(ii)-(iii) show ThT outcomes (colored traces) for individual quadruplet wells for the tested CSF dilution (ii, 1/22; iii, 1/25 in each case). Healthy CSFs tested in quadruplicate wells were used as controls. A(iv)-(v), B(iv)-(v), and C(iv)-(v) show example ThT outcomes for quadruplet wells for tested healthy CSFs. In each case, ThT threshold (horizontal broken line) and time cut-off (vertical broken line) for deciding positive/negative wells are shown. (DOCX) [file ppat.1012554.s010.docx]

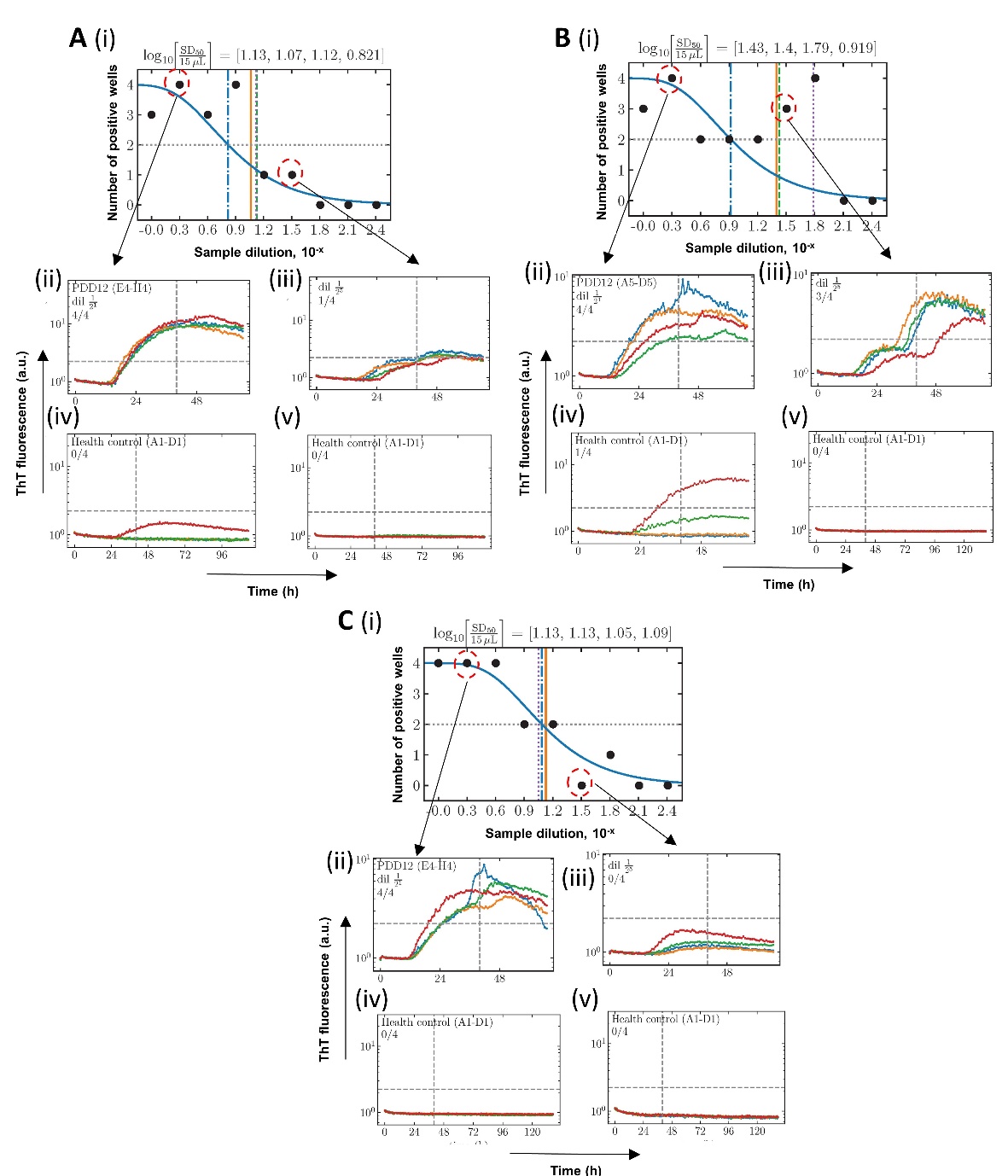


**S10 Fig.** Variability of outcomes and noise in CSF ED assays. A(i)-C(i) Outcomes from three independent 2F4R ED assays performed separately for CSF dilutions (PD6) are shown. Black arrows show ThT outcome at two different dilutions (broken red circles, 1/2^2^and1/2^5^) tested in 3 independent ED assays. A(ii)-(iii), B(ii)-(iii)**,** and C(ii)-(iii) show ThT outcomes (colored traces) for individual quadruplet wells for the tested CSF dilution (ii, 1/2^2^; iii, 1/2^5^ in each case). Healthy CSFs tested in quadruplicate wells were used as controls. A(iv)-(v), B(iv)-(v)**,** and C(iv)-(v) show example ThT outcomes for quadruplet wells for tested healthy CSFs. In each case, ThT threshold (horizontal broken line) and time cut-off (vertical broken line) for deciding positive/negative wells are shown.
