# Supplementary material for: Enhanced quantitation of pathological α-synuclein in patient biospecimens by RT-QuIC seed amplification assays
Source: PLoS Pathog. 2024 Sep 20;20(9):e1012554. doi: 10.1371/journal.ppat.1012554 (PMC11451978; doi:10.1371/journal.ppat.1012554)
Supplement: S11 Fig — Outcomes from three independent ED assays performed on skin samples from (A)-(D) two PD patients; (E)-(F) one DLB patient; and (G)-(H) one OM sample from a PD patient, each performed using either the 10F4R (A, C, E, G) or 2F8R (B, D, F, H) assay layout. The graphs are as described in the caption of S4 Fig (A,C). The mg denominators in (A)-(F) refer to weight of solid tissue and the μL denominators in (G)-(H) refer to volume of packed OM swabbing-derived pellet. (DOCX) [file ppat.1012554.s011.docx]

**
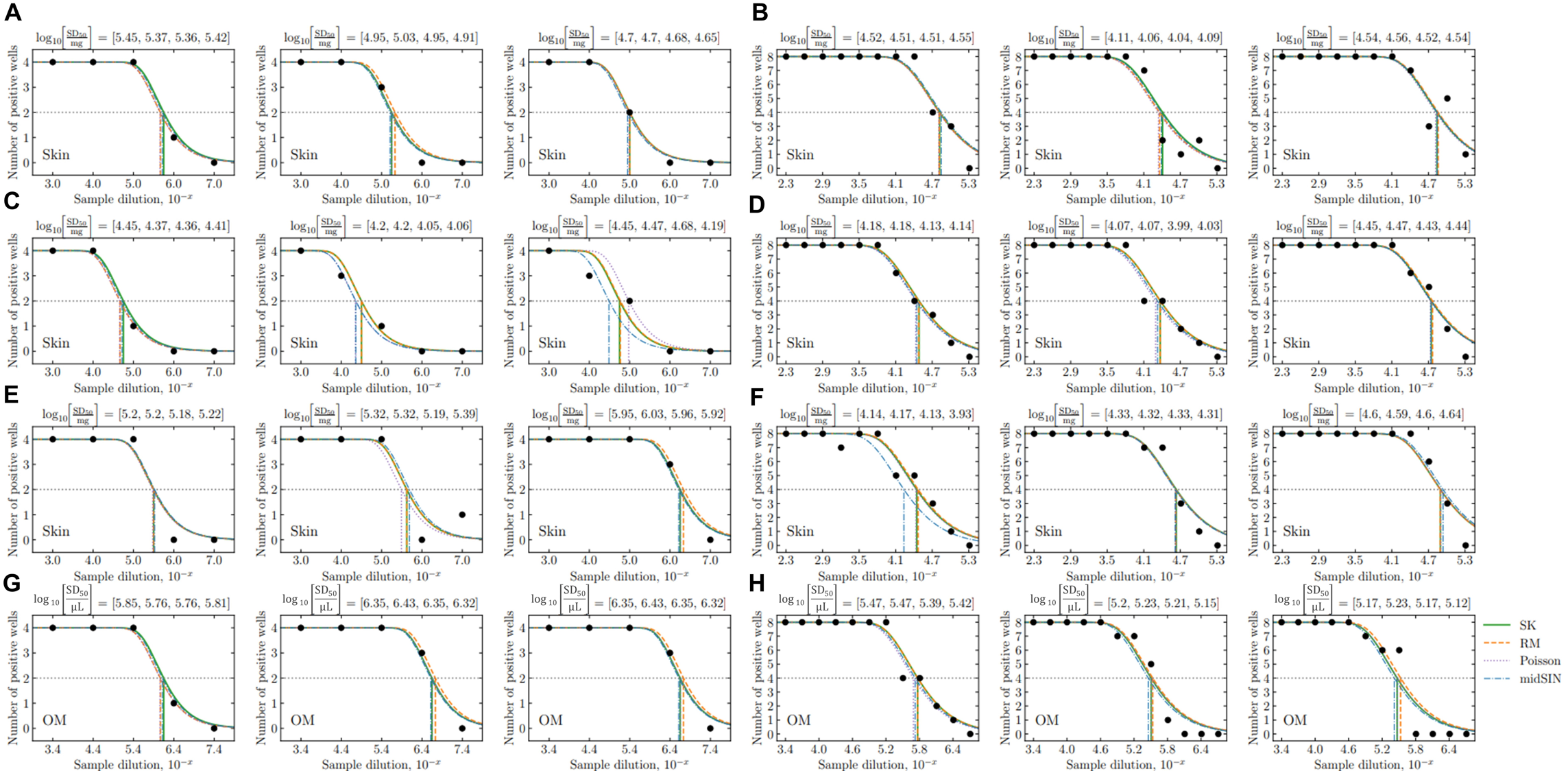
**

**S11 Fig.** Assessment of 10F4R and 2F8R ED assays in quantifying αSyn^D^ seeding Skin and OM samples from PD and DLB patients. Outcomes from three independent ED assays performed on skin samples from (A)-(D) two PD patients; (E)-(F) one DLB patient; and (G)-(H) on one OM sample from a PD patient, each performed using either the 10F4R (A, C, E, G) or 2F8R (B, D, F, H) assay layout. The graphs are as described in the caption of S4 Fig (A,C). The mg denominators in (A)-(F) refer to weight of solid tissue and the µL denominators in (G)-(H) refer to volume of packed OM swabbing-derived pellet.
