# Supplementary material for: Enhanced quantitation of pathological α-synuclein in patient biospecimens by RT-QuIC seed amplification assays
Source: PLoS Pathog. 2024 Sep 20;20(9):e1012554. doi: 10.1371/journal.ppat.1012554 (PMC11451978; doi:10.1371/journal.ppat.1012554)
Supplement: S1 Table — (DOCX) [file ppat.1012554.s012.docx]

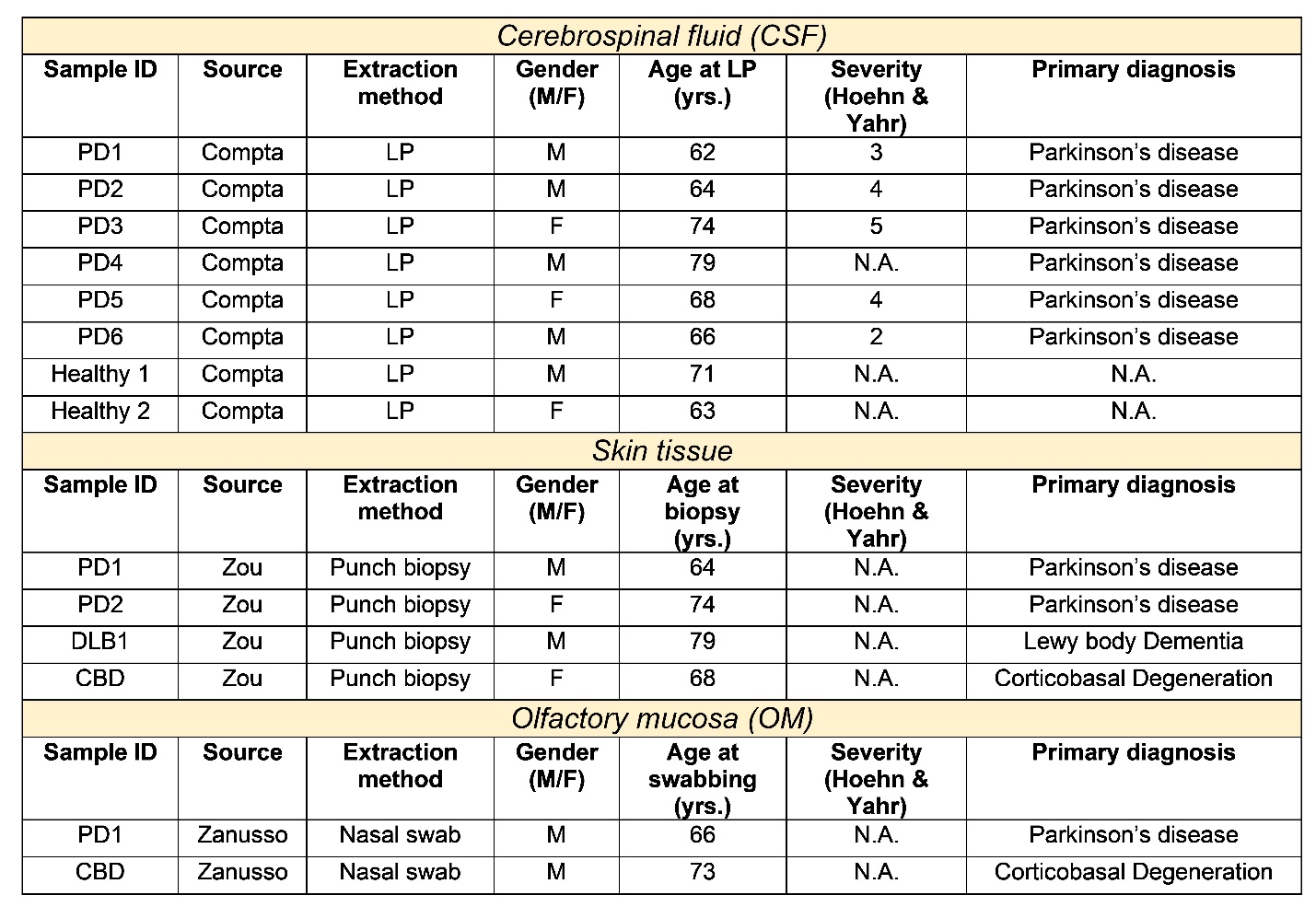


**S1 Table.** Table showing clinical and neuropathological characteristics of synucleinopathy and non-synucleinopathy CSF, skin and OM were used in the study.
